# Land-Bridge Calibration of Molecular Clocks and the Post-Glacial Colonization of Scandinavia by the Eurasian Field Vole *Microtus agrestis*

**DOI:** 10.1371/journal.pone.0103949

**Published:** 2014-08-11

**Authors:** Jeremy S. Herman, Allan D. McDevitt, Agata Kawałko, Maarit Jaarola, Jan M. Wójcik, Jeremy B. Searle

**Affiliations:** 1 Department of Natural Sciences, National Museums Scotland, Edinburgh, United Kingdom; 2 Mammal Research Institute, Polish Academy of Sciences, Białowieża, Poland; 3 Statistical Office, Centre for Forestry and Preservation of Nature, Białystok, Poland; 4 Department of Clinical and Experimental Medicine, Linköping University, Linköping, Sweden; 5 Department of Ecology and Evolution, Cornell University, Ithaca, New York, United States of America; Institute of Biochemistry and Biology, Germany

## Abstract

Phylogeography interprets molecular genetic variation in a spatial and temporal context. Molecular clocks are frequently used to calibrate phylogeographic analyses, however there is mounting evidence that molecular rates decay over the relevant timescales. It is therefore essential that an appropriate rate is determined, consistent with the temporal scale of the specific analysis. This can be achieved by using temporally spaced data such as ancient DNA or by relating the divergence of lineages directly to contemporaneous external events of known time. Here we calibrate a Eurasian field vole (*Microtus agrestis*) mitochondrial genealogy from the well-established series of post-glacial geophysical changes that led to the formation of the Baltic Sea and the separation of the Scandinavian peninsula from the central European mainland. The field vole exhibits the common phylogeographic pattern of Scandinavian colonization from both the north and the south, however the southernmost of the two relevant lineages appears to have originated *in situ* on the Scandinavian peninsula, or possibly in the adjacent island of Zealand, around the close of the Younger Dryas. The mitochondrial substitution rate and the timescale for the genealogy are closely consistent with those obtained with a previous calibration, based on the separation of the British Isles from mainland Europe. However the result here is arguably more certain, given the level of confidence that can be placed in one of the central assumptions of the calibration, that field voles could not survive the last glaciation of the southern part of the Scandinavian peninsula. Furthermore, the similarity between the molecular clock rate estimated here and those obtained by sampling heterochronous (ancient) DNA (including that of a congeneric species) suggest that there is little disparity between the measured genetic divergence and the population divergence that is implicit in our land-bridge calibration.

## Introduction

Phylogeography makes use of spatially ordered genetic data for biogeographical inference. Time is fundamentally important to any phylogeographic interpretation, so the rate of genetic change must be assumed or inferred in some way. Appropriate calibration can be accomplished by including genetic data from temporally spaced sequences [1], for example ancient DNA [2], [3]. Unfortunately, intraspecific phylogeographic studies often rely on the application of published molecular clock rates, as in previous phylogeographic studies of northern Eurasian *Microtus* voles [4]–[6]. These rates are generally derived from the genetic divergence between clearly-defined taxa whose separation can be dated from fossil evidence. This will introduce considerable uncertainty, both from the process of geological dating and the incomplete nature of the fossil record itself [7]. However, it is also inappropriate to apply such rates to recent genetic divergence among populations, given that they have been derived from fixed differences that have accumulated over millions of years [8]. It has for some time been recognised that there is a decay in molecular evolutionary rates, when these are measured over increasing periods of time [9], [10]. This phenomenon has generally been attributed to the effects of genetic drift and purifying selection on deleterious mutations [10], [11]. Whatever its origin, there is growing realization of the need to take account of time-dependence in molecular rate estimates [9], [12], as the application of clock rates derived from fixed genetic distances between species to intraspecific genetic data can lead to errors in orders of magnitude [8]. Evidence for time dependence and its influence on the inferred timing of evolutionary events are outlined in a recent review [10].

These contentious issues were fundamentally important to an earlier analysis of range-wide mitochondrial cytochrome *b* variation in the Eurasian field vole [13], which is one of three incipient or closely related species that make up the *Microtus agrestis* complex [14]. In that study, an extremely high nucleotide substitution rate (ca.4×10^−7^ substitutions/site/year) was obtained, around 20 times faster than a commonly used mammalian mitochondrial clock rate [15]. The rate was inferred by calibrating the gene genealogy from the possible time of origin of a monophyletic clade, currently restricted to northern Britain. It was assumed that the clade originated after 14.685 ka BP, when the climate rapidly warmed after the last Weichselian glaciation [16], as field voles would not tolerate the periglacial conditions that were present in north-western Europe before this time [17]. In addition it could not have originated more recently than 8.4 ka BP, the time when the island of Britain was separated from mainland Europe by the rising sea level of the Holocene [18] or it would form a subclade within a larger British clade.

This calibration could of course be incorrect, if the clade survived through one or more of the Weichselian stadials, either *in situ* or in a more southerly location with a more hospitable climate. Survival *in situ* is a possibility, because our assumptions about the species' climatic tolerance are based on its response to the present-day combination of environmental factors and biota. Survival of this clade elsewhere is also possible, but there is no evidence for this, despite the intensive sampling of adjacent parts of Europe [13]. Previous survival of the clade elsewhere would imply large-scale replacement of the lineage over its original range, breaking the link between past and present distributions of populations. The calibration that we actually use not only has the obvious correspondence between population divergence and environmental change, it also implies an association of genetic and population divergence. Genetic divergence will generally precede population divergence and the disparity between them may be considerable, especially in large populations. It is therefore important to acknowledge the possibility that sampled variation may represent persistent polymorphism that was present at the time of population divergence [11].

Although the field vole cytochrome *b* genealogy of Herman and Searle [13] was calibrated from the time of origin of a clade confined to northern Britain, within that genealogy there was also another monophyletic clade with a restricted distribution in the southern part of the Scandinavian peninsula. The sequences representing this clade had previously been incorporated within one of two widespread lineages that were thought to have colonized Fennoscandia from two separate centers in mainland Europe and Russia [6], [19], [20]. The field vole mitochondrial genetic structure therefore appeared to follow the general pattern of bi-directional immigration from the north-east and south that has been observed in other small mammals, such as the common shrew *Sorex araneus* and root vole *Microtus oeconomus*, as well as in other animals and plants [4], [6], [20]–[23].

However, based on the few Scandinavian samples available at the time, our more recent analysis [13] showed that the southern part of the Scandinavian peninsula was occupied by a distinct clade that was not present elsewhere and which originated at the same time as the five other clades that together make up the whole cytochrome *b* genealogy. This is in contrast to other species where the clade(s) occupying the Scandinavian peninsula were also present in central or eastern Europe [4], [22], [23]. For the field vole, one of the two source populations for the bi-directional Fennoscandian colonization had therefore originated in eastern Europe or western Asia, while the other was confined to the southern part of the Scandinavian peninsula itself. Field voles with a distinct Y-chromosomal re-arrangement (*Lund-Y*) are confined to one area within the southern part of the peninsula [24], [25], providing further evidence that a distinct lineage may have evolved here.

For the present study, we obtained 136 new field vole cytochrome *b* sequences from Scandinavia, Poland and Russia. The addition of substantial new data from eastern Europe and Scandinavia has allowed us to build on the earlier range-wide analysis of Herman and Searle [13]. We have reduced the heavy bias towards the British Isles in our previous sampling regime, while including substantial data for the geographical region that allows a second calibration of the genealogy (Scandinavia). The use of another calibration, similar to the previous one from Britain, permits us to refine our understanding of post-glacial colonization by the field vole and test our previous estimate of an intraspecific cytochrome *b* substitution rate. This new calibration is more certain than the previous one, as the southern part of the Scandinavian peninsula was entirely glaciated. Although it is vulnerable to the same risk as for the isolated British lineage, that the southern Scandinavian lineage survived one or more of the Weichselian stadials in an undetected mainland European refugium, it would be surprising if both isolated lineages were completely replaced in mainland Europe without any trace being found. Although our new calibration remains vulnerable to the possible presence of persistent polymorphism, pre-dating the divergence of the Scandinavian population, the otherwise low risk of calibration error permits us to reliably compare the result here with similar findings from studies that have employed heterochronous (ancient DNA) samples.

Some degree of discordance is expected between individual gene genealogies, like the one here, and their corresponding species trees [26]–[28]. However, in a study of speciation in the whole Eurasian field vole (*sensu lato*) complex [14], it was found that the distributions of mitochondrial lineages matched those defined by autosomal and sex-chromosome markers. Although the depth of corresponding nodes varied among the constituent genealogies, much finer resolution was obtained with cytochrome *b* than with the nuclear markers, due to the higher variability of cytochrome *b*. The exclusive use of mitochondrial DNA here is therefore justified, given the need for sufficient resolution and unbiased estimation of the field vole cytochrome *b* substitution rate. It permits direct comparison between the rate obtained here and those from other studies that have used heterochronous sampling, which have so far generally been restricted to mitochondrial DNA.

### Scandinavia at the end of the last glaciation

**Figure 1:**
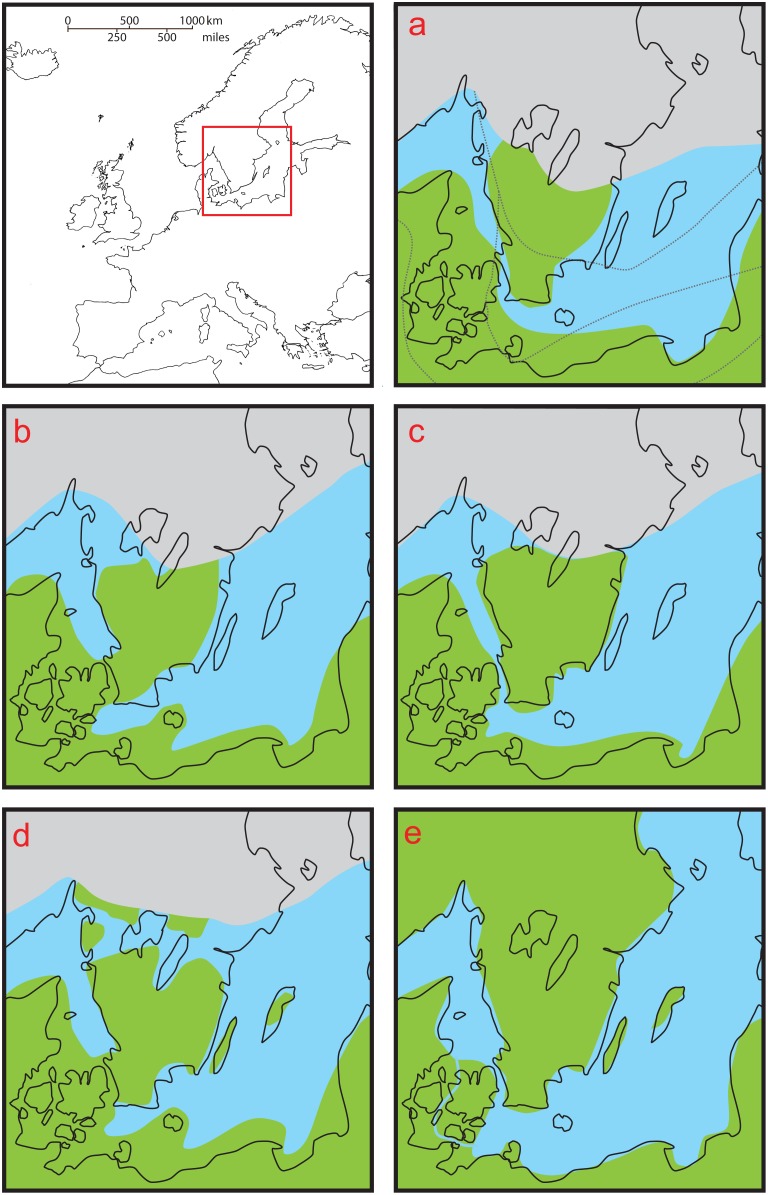
Stages of land connectivity relating to mammal colonization of Scandinavia. Based on reconstructions in Björck [30], [31]; see text for detail. Dates in ka BP, defined as thousand years before AD 1950, calibrated using IntCal04 and IntCal09 curves [32], [33]. Fennoscandian ice sheet - light gray, land - green, open water - light blue, modern coastline - black outline. **a:** Deglaciation of Scandinavian peninsula, until 13.1 ka BP (with dotted lines showing successive positions of ice sheet edge during late glacial retreat); **b:** First land bridge, 13.1-12.7 ka BP; **c:** Younger Dryas glacial re-advance and re-opening of Øresund channel, 12.7-12.1 ka BP; **d:** Second land bridge, 12.1-10.3 ka BP; **e:** Separation of Scandinavian peninsula by Dana River and then formation of Baltic Sea, 10.3-9.2 ka BP.

In Scandinavia and the Baltic region of north-eastern Europe, the episodes of climate change drove a complex but well-established process of geophysical change that was dominated by the deglaciation of the Fennoscandian ice-sheet [29]. Eustatic and isostatic changes in sea level produced a dynamic pattern of connectivity between land masses in the region [30], [31] and this can conveniently be divided into a series of discrete, timed stages that are relevant to colonization by small mammals and other terrestrial animals (Figure 1). For consistency all dates quoted here are in ka BP, defined as thousand years before AD 1950, with ^14^C values calibrated using the IntCal04 and IntCal09 curves [32], [33].

Until 13.1 ka BP the Scandinavian and Baltic region was deglaciating (Figure 1a), but the ice cover of the southern Scandinavian peninsula was replaced by Arctic tundra [34], unsuitable for temperate species like the field vole and furthermore inaccessible to animals that could not use winter ice cover to cross the Baltic Ice Lake outlet in the Øresund area. From 13.1 to 12.7 ka BP, the Baltic Ice Lake drained sub-glacially at the margin of the receding ice-sheet and a first land bridge was formed between the southern part of the Scandinavian peninsula and mainland Europe (Figure 1b). This land bridge, together with the prevailing warmer climate, surely provided the first opportunity for temperate small mammals to colonize the Scandinavian peninsula from the south. Between 12.7 and 12.1 ka BP, the Øresund outlet of the Baltic Ice Lake was re-established and the land bridge was no longer available, because the cooling temperature of the Younger Dryas period brought the retreat of the ice-sheet to a halt and closed the sub-glacial outflow (Figure 1c). A second land bridge connected the southern part of the Scandinavian peninsula to mainland Europe from 12.1 to 10.3 ka BP, once again providing a route for colonization by small mammals. This land-bridge was formed by the re-opening of a northern outlet for the Baltic Ice Lake, as the ice-sheet began to retreat again (Figure 1d). During this period the Baltic Ice Lake was finally drained and replaced by the Yoldia Sea, then the Ancylus Lake, when the northern outlet was dammed again, this time by uplift of southern Scandinavia as the Fennoscandian ice-cap melted. At 10.3 ka BP the Dana River was formed to the west and south of Zealand, as the continuing uplift of the southern Scandinavian peninsula reduced and eventually closed the northern outlet of the Ancylus Lake through Lake Vänern, then at 9.2 ka BP the Baltic Sea was created by inundation of the Øresund channel (Figure 1e). At this point the Scandinavian peninsula was finally separated from mainland Europe, preventing further overland colonization from the south.

## Materials and Methods

Genomic DNA was extracted from 136 preserved skins of field voles from Norway, Poland and Russia. These were obtained from the collections of the Mammal Research Institute, Polish Academy of Sciences, Białowieża, University Museum of Bergen, Norway and National Museums Scotland, Edinburgh (Table S1). The entire 1 143 base-pair cytochrome *b* gene was amplified using the primers in Table S2 and a protocol designed for this type of material [35], [36]. Museum skins yield reduced amounts of DNA that also tends to be fragmented [37], so the gene was amplified in four overlapping fragments of 300 to 400 base-pairs (Figure S1). Negative extraction and PCR controls, with no tissue and no template DNA respectively, were included in all procedures. Sequences were edited and aligned with 305 previously published sequences from the Palaearctic range of the species [6], [13], [38]. All new sequences are available in the GenBank database (KF218851–KF218952) and voucher specimens in the three institutions above (Table S1).

Bayesian genealogy sampling with BEAST 1.7.5 [39] was used to infer the relationship among the cytochrome *b* sequences, represented here by the Maximum Clade Credibility Tree, along with times to most recent common ancestor (tMRCA) for the main clades. Posterior distributions of these and other model parameters were obtained from four or more independent Monte Carlo Markov chain (MCMC) simulations, each run for 200 million generations, by which time the effective sample size for each parameter was sufficient (200 or more). Convergence of chains was confirmed from log traces for each parameter and the first 10 million generations from each chain were discarded as burnin. The analyses were repeated without sequence data, to test the effects of the priors and the data on the posterior distributions.

Simulations were carried out with a variety of potentially appropriate coalescent models. A strict molecular clock was compared with an uncorrelated lognormal (UCLN) relaxed molecular clock [40], which is commonly used to accommodate variation in clock rate across the branches of the genealogy. Constant population size, simple expansion growth and skyline demographic models [41] were also compared, to test whether the population size had changed and attempt to recover the pattern of change, where present. Path sampling and stepping-stone sampling were used to estimate marginal likelihoods for comparisons between these models [42]. For model selection, MCMC runs comprised 1 000 steps of 100 000 generations, with power posteriors defined according to quantiles of a beta distribution with alpha value 0.3, following 10 million generations burnin. Sufficient sampling of chains was indicated by convergence of the combined marginal likelihood estimate from path sampling runs with that from stepping-stone sampling runs, which is attained more rapidly. In preliminary tests of the method, convergence was much more rapid under this regime of many short chains than when regimes with fewer steps and longer chains were used (100 steps of 1 000 000 generations and 50 steps of 2 000 000 generations). Proper prior distributions were specified for all parameters, a requirement for estimation of marginal likelihoods. The resulting Bayes Factors, expressed as natural logarithms, for preference of one model over the other were interpreted according to widely accepted criteria [43].

The molecular clock was calibrated using prior distributions on the tMRCA of the clade from the southern part of the Scandinavian peninsula, together with the root height. The tMRCA of the southern Scandinavian clade was given a normal distribution truncated at lower and upper limits of 13.1 ka and 9.2 ka BP, respectively. The earlier date represents the first opportunity for a temperate species to colonize the southern part of the Scandinavian peninsula, which was until then Arctic tundra separated from mainland Europe by the newly forming Baltic Ice Lake (Figure 1a). The more recent limit reflects the assumption that the clade could not have originated after 9.2 ka BP, the time when the Scandinavian peninsula was finally separated from mainland Europe (Figure 1e), or it would form a subclade within a larger Scandinavian clade. The root height was given a gamma distribution that peaked around 145 ka BP, which we considered the most plausible date within the 95% confidence limits of a previously estimated time of divergence for the whole species [6], given that it coincides with the end of the penultimate (Saalian) glacial period. The evidence for this date is limited, so its influence was tested by running simulations with gamma distributions that peaked at around 24 ka BP and 450 ka BP, coinciding with the final maxima of the last (Weichselian) glaciation and that of the Elsterian glaciation, which immediately preceded the Saalian glacial period. In addition, the minimum of the root height distribution was placed at 14.685 ka BP, the time of rapid warming that marked the end of the most recent (Weichselian) glaciation [16]. We constrained the root of the tree, representing the common ancestor of the whole extant field vole population, to pre-date this event on the grounds that the whole species would not be subject to such an extreme population bottleneck after the last full glacial period.

Summary statistics were also calculated, to quantify genetic variation in each of the six lineages that were identified by genealogy sampling. Nucleotide diversity, average number of substitutions per site between all sequence pairs, was estimated using MEGA 5.05 [44]. The calculation used the Kimura 2-parameter nucleotide substitution model and gamma distribution of rates across sites, with an alpha shape parameter of 0.1705 estimated using maximum likelihood. Standard errors were estimated from 1 000 bootstrap replicates and t-tests were used to determine the significance of any difference between groups. Two neutrality test statistics, Tajima's *D* and Fu's *F_S_*, were estimated with DnaSP 5.10.01 [45]. These can be seen as an indicator of recent population expansion, which leads to an excess of singleton mutations in external branches of the phylogeny. The significance of any departure from neutrality was determined by comparing the value of the test statistic with an empirical distribution obtained by randomly placing the observed number of mutations onto 10 000 coalescent simulations of the genealogy.

## Results

There were 304 distinct haplotypes among the 441 sequences available from this study and GenBank (Table S1). Similar results were obtained for genealogy sampling trials with a range of GTR-nested nucleotide substitution models and those reported here were obtained using a 2-partition (first and second codon position linked; third separate) HKY substitution model, as recommended for protein-coding nucleotide data [46], with gamma distribution of rates among sites. The marginal likelihoods from path-sampling indicated that the relaxed UCLN clock was very strongly favoured over the strict clock (log_e_ Bayes Factor = 5.6). This is rather surprising for intraspecific data but reflected in the posterior distribution of the standard deviation of the relaxed clock rate, which peaked above zero, although its lower tail nevertheless abutted the origin.

**Figure 2:**
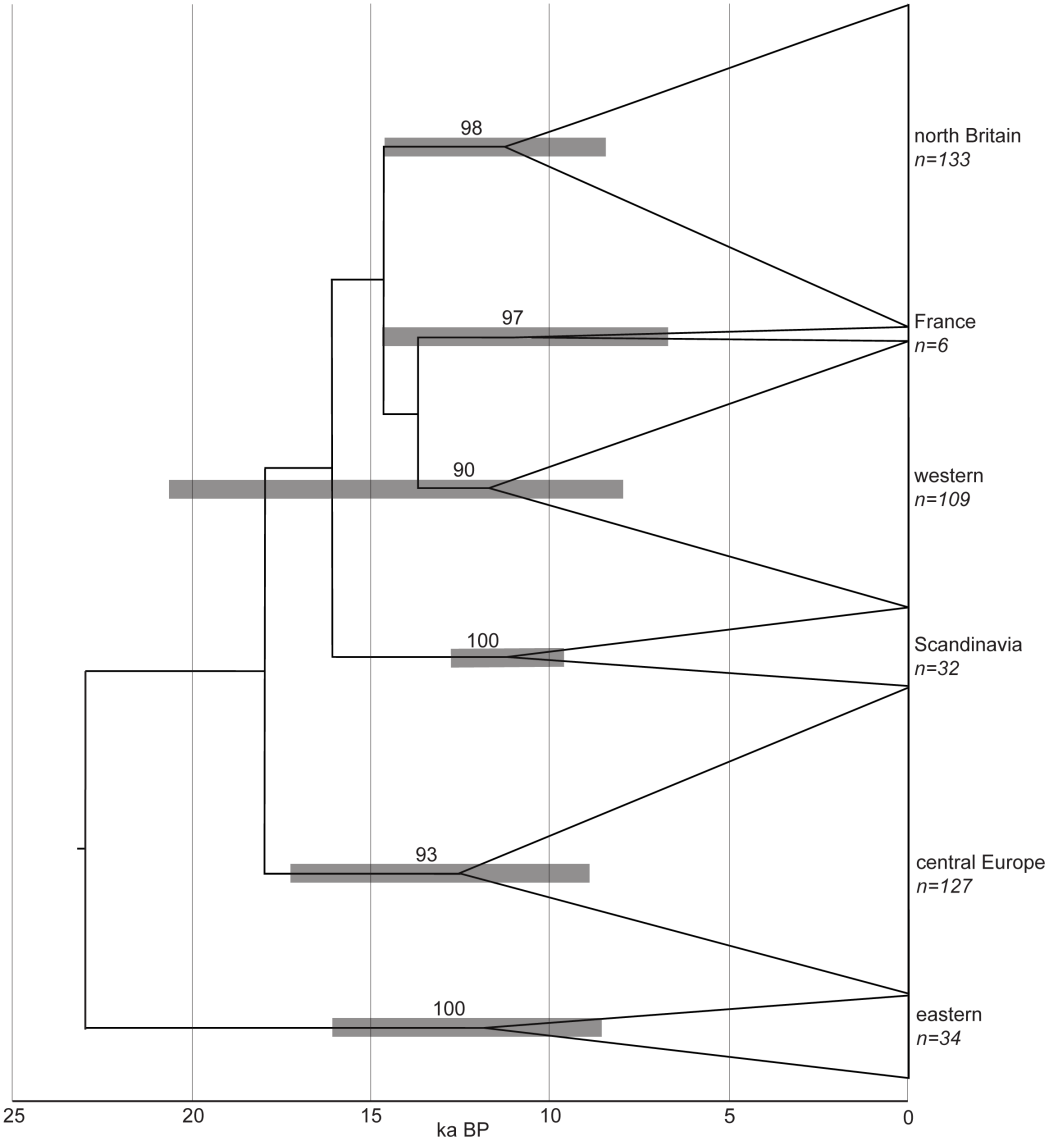
Field vole mitochondrial cytochrome *b* genealogy. Maximum clade credibility tree from Bayesian coalescent modelling with 441 sequences, clade support from posterior probability of node. Clades collapsed for clarity and gray bars show 95% highest posterior density intervals for tMRCA of each lineage.

**Figure 3:**
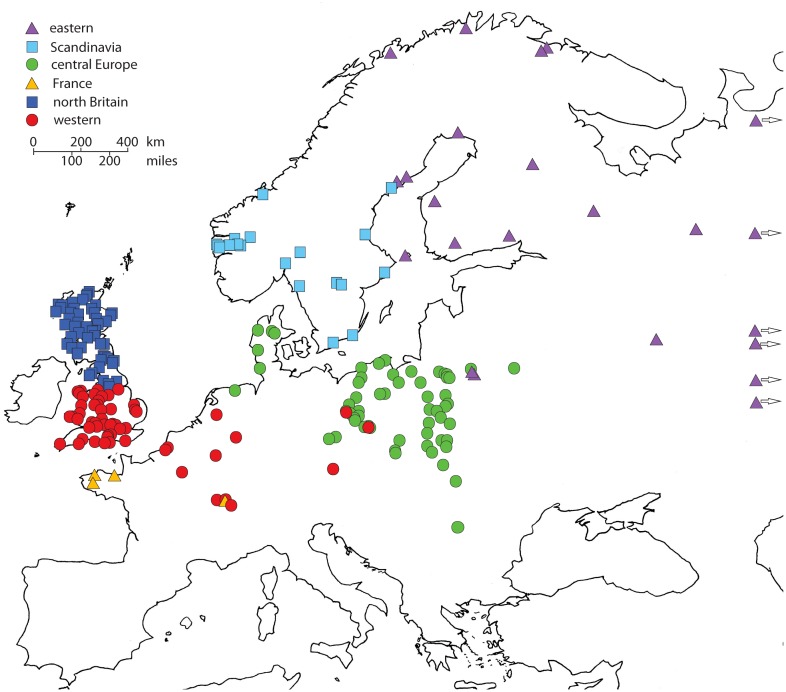
Geographical locations of samples from each clade. Closely adjacent localities assigned to same map location.

The 441 cytochrome *b* sequences were grouped into six well-supported lineages (Figure 2), named as *eastern*, *Scandinavia*, *central Europe*, *France*, *north Britain* and *western* following the previous results of Herman and Searle [13]. The new sequences from southern Scandinavia all belong to the previously-described lineage and no sequences from the *Scandinavia* clade were found in mainland Europe, despite substantial numbers of samples obtained there (Figure 3). The restricted distribution of the *Scandinavia* clade is of course critical to our land-bridge calibration of the genealogy.

The nucleotide diversities from each clade were similar (Table 1), as might be expected if they were of similar age. Although the *eastern* and *central Europe* clades appeared to be somewhat more variable than the others, which might reflect their large geographical ranges, none of the differences between clades were significant. The two test statistics for neutrality, Tajima's *D* and Fu's *F_S_*, have highly significant values for five of the six clade populations (Table 1). The only exception is the *France* clade, which may simply reflect the very small sample size available. The significant values for these statistics are consistent with recent demographic expansion. Taken together, these measures of genetic variation suggest that the six lineages originated from contemporaneous founding populations which have subsequently undergone marked growth.

**Table 1:** Genetic variation in the six mitochondrial lineages.

Lineage	*n*	*π*	se*_π_*	Taj *D*	P_coal_	Fu *F_S_*	P_coal_
eastern	34	0.00914	0.00142	−1.87491	0.01150	−26.691	0.00000
Scandinavia	32	0.00703	0.00107	−1.80783	0.01610	−14.506	0.00010
central Europe	127	0.00902	0.00132	−1.77186	0.01090	−89.497	0.00000
France	6	0.00686	0.00180	−0.32862	0.43300	1.967	0.81411
north Britain	133	0.00631	0.00115	−2.04066	0.00170	−86.386	0.00000
western	109	0.00722	0.00130	−2.09957	0.00150	−67.290	0.00000

Nucleotide diversity (*π*) with standard error estimated from 1 000 bootstrap replicates. Neutrality test statistics (Tajima's *D* and Fu's *F_S_*) with significance derived from 10 000 coalescent simulations.

Based on genealogy sampling with the new calibration from the root height and the southern Scandinavian clade, the whole of the existing species' population would appear to have originated from a single group of founders around 23 ka BP, the time of the last glacial maximum (LGM). According to the test runs with the two alternative prior distributions on the root height, the prior had little influence on posterior estimates of this parameter. The current mitochondrial genetic structure is derived from a group of six founding populations that originated around 12 ka BP, the time of the Younger Dryas glacial re-advance. There is some variation in the time of origin of the different clades (Table 2, Figure 2), which might relate to differences between the timing of climatic events in specific geographic regions, but the margins of error in the analysis do not allow any firm conclusions. The median tMRCA for the southern Scandinavian clade is 11.209 ka BP, which is well within the timing of the second, more recent, Scandinavian land bridge. However, the upper of the 95% HPD limits coincides with the presence of the earlier land bridge and much of the posterior distribution coincides with the Younger Dryas glacial re-advance (Figure 1c). Furthermore, the median tMRCA for the clade is very close to the mean of its prior distribution, despite the additional constraint that was placed on the root height.

**Table 2:** Times to most recent common mitochondrial ancestor (tMRCAs).

Lineage	95% HPD lower (ka BP)	Median (ka BP)	95% HPD upper (ka BP)
Root	16.477	22.933	32.185
eastern	8.480	11.835	16.073
Scandinavia	9.586	11.209	12.828
central Europe	8.878	12.444	17.197
France	6.724	10.512	14.722
north Britain	8.378	11.272	14.562
western	7.855	11.629	20.697

Time to most recent common mitochondrial ancestor (tMRCA) for whole field vole population and the six clades. Median and 95% highest posterior density (HPD) range of times, obtained with Bayesian genealogy sampling, calibrated with possible times of origin of whole species and clade from southern Scandinavian peninsula.

The relatively high molecular clock rate (mean 4.572×10^−7^ substitutions/site/year, 95% highest posterior density interval 3.411–5.834×10^−7^ substitutions/site/year) is close to the one that was obtained using a calibration from the clade confined to northern Britain, although it is based on this new calibration with a different clade.

**Figure 4:**
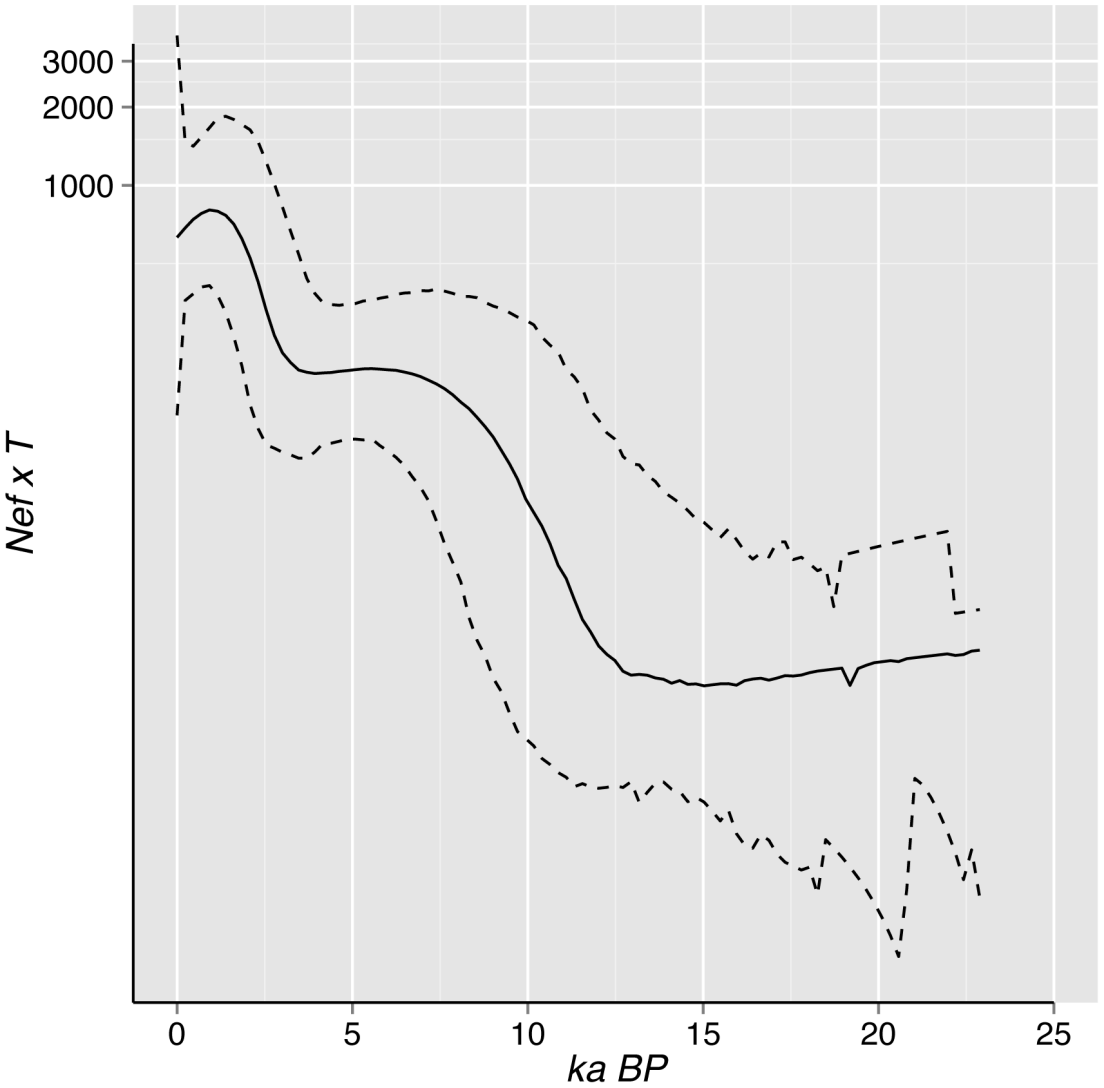
Bayesian skyline plots showing effective female population size. Effective female population size (*N_ef_*), in thousands, multiplied by mean generation time (*T*), in years. Heavy line is median and lighter lines are 95% highest posterior density (HPD) limits. *N_ef_ x T* plotted on log scale for clarity and truncated to median estimate of tMRCA.

The marginal likelihoods provided very strong evidence for the expansion growth model over the constant population size model (log_e_ Bayes Factor = 76.3) and for the Bayesian Skyline model over the simpler expansion model (log_e_ Bayes Factor = 30.5), indicating that the field vole population has undergone demographic expansion and that this can be more accurately recovered from these data using the skyline model. The Bayesian skyline plot, of effective female population size with time, is fairly flat until the origin of the six clades around the time of the Younger Dryas, at which point it shows a marked rise which flattens around 8 ka BP (Figure 4). There is therefore no sign of demographic change between the origin of the whole population at the LGM until the time when the six regional lineages began to expand after the Younger Dryas. The simplest explanation is that the whole of the species' current population is derived from a single group of founders that underwent a population bottleneck at the time of the LGM. The species must have colonized much of the western Palaearctic region during the subsequent Bølling–Allerød interstadial, for the six regional populations to have originated in different parts of its current range. However any signal of population growth would be wiped out by a second set of bottlenecks, at the time of the Younger Dryas, which apparently gave rise to these six lineages. The skyline plot also shows another recent demographic expansion, previously attributed to clearance of woodland by humans in north-western Europe [13].

It should be noted here that in the tests of genealogy sampling using the prior distributions alone, with no sequence data, the estimated root height was more recent than its prior median value, while the tMRCAs of the clades were roughly proportional to the number of sequences within them, up to the age of the root. This can be attributed to the relative constraints on these nodes in the genealogies. However it appears that this does not unduly alter the posterior parameter distributions that were estimated here, and therefore does not affect the conclusions drawn, as the root height inferred with priors alone was still markedly different from the age inferred with the data and the clade tMRCAs bore no relation whatsoever to the inferred ages.

## Discussion

In the absence of dated ancient DNA sequences, calibration of intraspecific data can be achieved by aligning common ancestors with contemporaneous external events, deriving from geological [47] or archaeological [48] evidence. The field vole is a potentially useful model for this process, because it has colonized parts of Europe that have experienced dramatic geophysical changes, for which there are precise and reliable reconstructions. This allows the putative alignment of population divergence and genetic variation with timed events that are either causative or affected by the same process of climate change. We have previously made use of geophysical events around the British Isles [13], but here we are able to test these earlier reconstructions with an independent and more certain calibration from similar events in Scandinavia.

With regard to the calibration itself, it is important to take account of the combined effect that is induced by associating priors with more than one node in the gene tree. Although the posterior of the calibration time will not be modified by the sequence data and will therefore be identical to its prior distribution [49], specification of multiple calibration priors will induce a joint prior, with a novel distribution, on the node heights [50]. It is therefore important to test the combined effect of the calibration priors and the sensitivity of the result. The marginal prior distributions of the clade tMRCAs and the root height, obtained by running the MCMC chains without sequence data, were somewhat different to their specified prior distributions. However, the posterior distributions of the tMRCAs that were obtained with sequence data were substantially different from their priors, indicating that they are not simply the product of the combined calibration priors. Meanwhile, the tMRCAs obtained with the three variants of the prior on root height were similar, suggesting that the posterior distributions of clade and root heights were generally dependent on the recent limit of the root height prior, which did not vary among the different prior distributions. Together, these results give us confidence that the calibration priors are not influencing the result in some unexpected way.

Our data show that the extant field vole population is made up from six mitochondrial lineages with coherent and generally allopatric geographical distributions of very different size (Figure 3). Based on the calibration here, the origins of the whole population and those of the six lineages can be associated with bottlenecks around the time of the LGM (23 ka BP) and Younger Dryas re-advance (12 ka BP) respectively. Bayesian genealogy sampling indicates overall demographic expansion from the single LGM population, as might be expected because the field vole must have colonized most of its present range since the last glaciation. Although the species must have colonized much of Eurasia beforehand, given the dispersed locations of the six lineages, these mitochondrial data show no sign of population expansion until the Younger Dryas when their demographic, and presumably spatial, expansion began. A high molecular clock rate (ca.4×10^−7^ substitutions/site/year) is obtained by calibration of the model from the clades confined to Scandinavia (here) or north Britain [13].

The southern and northern parts of the Scandinavian peninsula are occupied by members of two separate lineages and these two lineages have mutually exclusive distributions (Figure 3). The first of these lineages is confined entirely to southern Sweden and Norway, with no presence in mainland Denmark (Jutland) or mainland Europe, but the other lineage has a very wide distribution in north-eastern Europe and western Asia. It is generally accepted that the northern part of the Scandinavian peninsula was colonized from the north and east via Russia and Finland, following the last glaciation, and the southern part of the peninsula by means of a land bridge in the Øresund area between what are now Sweden and Denmark. This overall pattern of post-glacial Scandinavian colonisation has been inferred in various mammals, including the root vole *Microtus oeconomus* [4], [5], and other animals and plants [20]–[23]. The location of the resulting suture zone, running east-west across the middle of the peninsula, is likely due to the position of the last remnant of the Fennoscandian ice-sheet, which deglaciated in either direction towards this part of the peninsula, having retreated rapidly from north-western Russia and Finland at the beginning of the Holocene [29]. While the field vole appears to follow this general phylogeographic pattern within the Scandinavian peninsula [6], [19], our analysis here suggests that the lineage in the southern part of the peninsula originated either *in situ*, or possibly in what is now the Danish island of Zealand, around the close of the Younger Dryas. Zealand and the southern part of the Scandinavian peninsula were connected for a long period following the Younger Dryas, from 12.1 until 9.2 ka BP (see above; Fig. 1d,e), so field voles could have colonized the peninsula from Zealand at any time between these dates. Furthermore, there is evidence from mitochondrial RFLP data that field voles in Zealand and southern Sweden are closely related [19].

Radiocarbon dates from subfossil remains indicate that many mammal species entered the Scandinavian peninsula via the first post-glacial land bridge, or even earlier in a few cases, however almost all the large terrestrial species were lost from Sweden at the time of the Younger Dryas re-advance [51]. Most of the current mammalian fauna therefore colonized the Scandinavian peninsula across the more recent land bridge (Figure 1d). Although there appear to be no subfossil finds of field vole here before the Younger Dryas, it does seem likely that this species would closely follow the retreat of the ice and it has been recorded from the late Weichselian in Denmark [51]. The post-glacial expansion of the root vole seems to have followed a similar pattern to that of the field vole and there are remains of this species from the very late Pleistocene of both southern Sweden and Denmark [4]. Subfossil evidence from caves indicates that the field vole was also able to colonize the mainland of southern Britain before the Younger Dryas [52], and according to our data it appears to have survived the Younger Dryas there [13]. It therefore seems likely that the field vole would have colonized the southern part of the Scandinavian peninsula from northern mainland Europe during the original expansion of the single LGM refugial population over the first land bridge during the Bølling–Allerød interstadial (Figure 1b).

The lack of any fossil evidence for a Younger Dryas refugium in the southern Scandinavian peninsula is perhaps surprising, given that the field vole appears to have survived the glacial re-advance in southern Britain. However, the Scandinavian climate is more continental than that of the British Isles and there is likewise no sign of a distinct northern population in the adjacent eastern part of the species' range. On the contrary, a vast area of Russia and northern Fennoscandia was colonized from a single Younger Dryas refugium, presumably somewhere towards the southern limit of the area occupied by this lineage [6], [13]. Competition with tundra mammals presumably played a part in defining the northern limit of the species' distribution, in the eastern part its range. Although there do not appear to be any fossil remains of lemmings in southern Sweden from the time of the Younger Dryas [51], they may have survived the last glaciation in the northern part of the Scandinavian peninsula and have been recorded from mainland Europe at this time [53]. If tundra mammals were indeed part of the fauna, they would be in competition with any surviving field voles, when this isolated pocket in southern Fennoscandia returned to the colder conditions of the Younger Dryas. Nevertheless, it is difficult to reconcile the origin of the southern Scandinavian field vole lineage with any climatic event since the Younger Dryas.

Based on the analyses that we present here, the southern Scandinavian lineage does indeed appear to have originated *in situ* on the Scandinavian peninsula or in Zealand, probably around the end of the Younger Dryas. Although we have not found any members of this clade in mainland Europe, notwithstanding the number of samples sequenced from Poland and the Jutland region of Denmark, flawed assumptions about the distributions of populations are a potential source of calibration error in phylogeography. Nevertheless, the phylogeographic and demographic reconstruction that we obtain here is easily reconciled with the pattern of climatic and geophysical change at this time.

Calibrations from external events are equally vulnerable to the potential disparity between the timing of population divergence and that of the sampled genetic variation. The high molecular rates that we obtained with the Scandinavian data here, and with those from northern Britain, are therefore susceptible to error from the putative association of genetic and population divergence. However, they are very similar to a clock rate of 3.27×10^−7^ substitutions/site/year that was estimated from ancient DNA in the closely related common vole *Microtus arvalis* [54]. They are also consistent with mitochondrial clock rates that have been estimated from heterochronous samples in a wide range of other vertebrates [2], [55]. Rates like these are highly controversial, particularly when based on ancient DNA [56], so it is interesting that similar results are obtained with land bridge calibration here.

In view of the concordance between the rate estimated here and others obtained with ancient DNA, including that from *M. arvalis* [54], it appears that the assumptions inherent in our landbridge calibration were in this case justified. Our initial assumption, that population divergence relates to geophysical and climatic events, is hardly contentious, given the nature of these changes in northern Eurasia. However, it is more important that the result supports the close alignment of population divergence, as represented by the landbridge proxy, and (mitochondrial) genetic divergence. This finding suggests that the geographical distribution of genetic variation can indeed be used to infer the relationship between climatic change and the recent history of populations of species. It is therefore relevant to the controversial subject of time dependence in molecular rates, which is very important for understanding recent evolutionary events [10], and highlights the need for more thorough investigation of the molecular evolutionary processes on which phylogeographic inference rests. Indeed, the benefits of more genetic data and the integration of other evidence have been emphasized before [57], [58], given the perpetual and rapid nature of environmental change and the individual response it engenders in species and their populations.

The congruence between our result and those from heterochronous (ancient DNA) sampling suggest that mitochondrial genetic variation can be related to recent geophysical and climatic events, whose likely effects on historical populations are fairly certain. However, this does not imply that current phylogeographic methods are necessarily able to recover the history of deeper evolutionary changes. Although the use of multiple markers can resolve the potential problem of inconsistency between gene genealogies and species trees [26]–[28], it will not address the fundamental problem of how to distinguish between patterns that have arisen by dispersal and vicariance [59]. While the use of limited numbers of additional markers may be sufficient for intraspecific analyses, like the one here, it is likely that deeper and wider biogeographical insights will require the resolution of genomic data and more sophisticated analysis, perhaps involving other types of data.

## Supporting Information

Figure S1 **Primers for cytochrome b amplification.** Schematic representation of the relative position of the primers used to amplify the complete cytochrome *b*. The primer sequences are listed in Table S2.
(TIF)

Table S1 **Provenance of field vole cytochrome** ***b*** **sequences.** Vouchers are held in Mammal Research Institute, Polish Academy of Sciences, Białowieża (prefix MRI.PAS.), Bergen University Museum, Norway (prefix Berg.) and National Museums Scotland, Edinburgh (prefix NMS.Z.). New sequences *.
(DOCX)

Table S2 **Primers for cytochrome** ***b*** **amplification.** Primers used for PCR amplification and sequencing of cytochrome *b* gene in *Microtus agrestis* (see Figure S1 for approximate positions of primers).
(DOCX)
