# Supplementary figures and images for: Land-Bridge Calibration of Molecular Clocks and the Post-Glacial Colonization of Scandinavia by the Eurasian Field Vole Microtus agrestis
Source: PLoS One. 2014 Aug 11;9(8):e103949. doi: 10.1371/journal.pone.0103949 (PMC4128820; doi:10.1371/journal.pone.0103949)

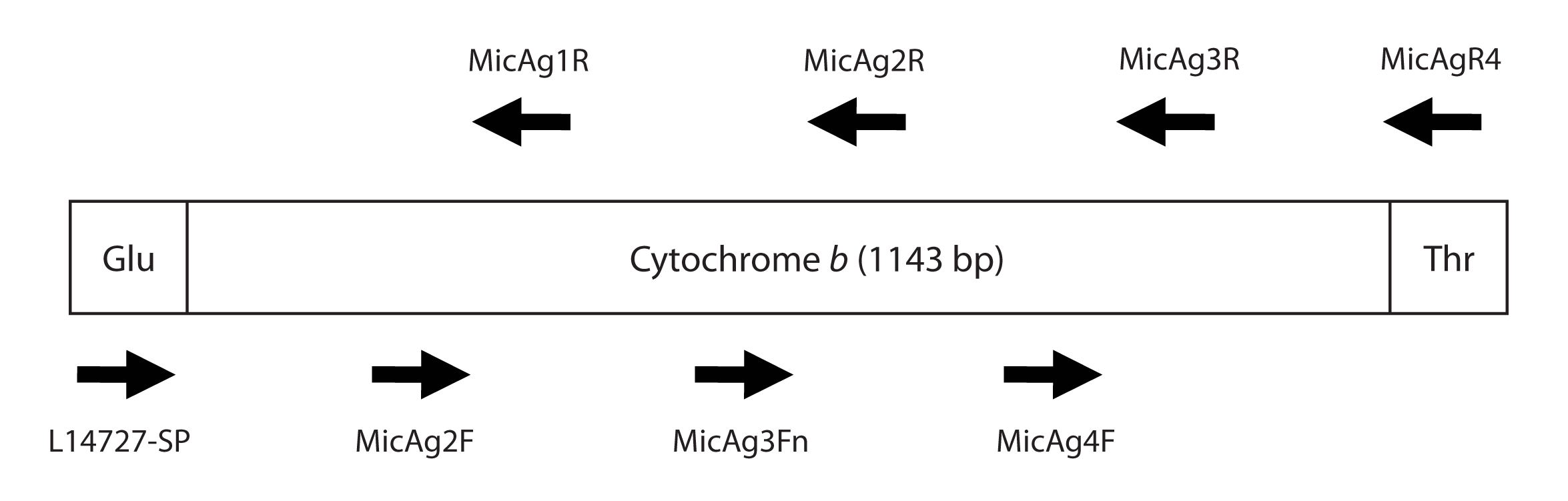

Supplement: Figure S1 — Primers for cytochrome b amplification. Schematic representation of the relative position of the primers used to amplify the complete cytochrome b. The primer sequences are listed in Table S2. (TIF) [file pone.0103949.s001.tif]
