# Supplementary material for: Land-Bridge Calibration of Molecular Clocks and the Post-Glacial Colonization of Scandinavia by the Eurasian Field Vole Microtus agrestis
Source: PLoS One. 2014 Aug 11;9(8):e103949. doi: 10.1371/journal.pone.0103949 (PMC4128820; doi:10.1371/journal.pone.0103949)
Supplement: Table S1 — Provenance of field vole cytochrome b sequences. Vouchers are held in Mammal Research Institute, Polish Academy of Sciences, Białowieża (prefix MRI.PAS.), Bergen University Museum, Norway (prefix Berg.) and National Museums Scotland, Edinburgh (prefix NMS.Z.). New sequences *. (DOCX) [file pone.0103949.s002.docx]

**Table S1. Provenance of field vole cytochrome *b* sequences.**

Vouchers are held in Mammal Research Institute, Polish Academy of Sciences, Białowieża (prefix MRI.PAS.), Bergen University Museum, Norway (prefix Berg.) and National Museums Scotland, Edinburgh (prefix NMS.Z.). New sequences *.

| **Location** | **Latitude** | **Longitude** | **Lineage** | **GenBank** | **Voucher/source** |
| --- | --- | --- | --- | --- | --- |
| Belarus, Berezina | 54.6667 | 28.5000 | central Europe | AY167155 | Jaarola and Searle 2002 |
| Belarus, Berezina | 54.6667 | 28.5000 | central Europe | AY167155 | Jaarola and Searle 2002 |
| Belarus, Berezina | 54.6667 | 28.5000 | central Europe | AY167155 | Jaarola and Searle 2002 |
| Czech Republic, Ústecký kraj, Filipov | 50.8167 | 14.3833 | central Europe | AY167152 | Jaarola and Searle 2002 |
| Czech Republic, Ústecký kraj, Fláje | 50.6833 | 13.5667 | central Europe | AY167151 | Jaarola and Searle 2002 |
| Czech Republic, Jihočeský kraj, Kladenské Rovne | 48.8000 | 14.2833 | western | AY167212 | Jaarola and Searle 2002 |
| Czech Republic, Jihočeský kraj, Kladenské Rovne | 48.8000 | 14.2833 | western | AY167212 | Jaarola and Searle 2002 |
| Denmark, Ringkjøbing, Hjerl Hede | 56.4333 | 8.4167 | central Europe | AY167179 | Jaarola and Searle 2002 |
| Denmark, Ringkjøbing, Hjerl Hede | 56.4333 | 8.4167 | central Europe | AY167184 | Jaarola and Searle 2002 |
| Denmark, Viborg, Ulstrup | 56.3833 | 9.7833 | central Europe | AY167194 | Jaarola and Searle 2002 |
| Denmark, Aarhus, Emilsminde | 56.2500 | 10.0000 | central Europe | AY167179 | Jaarola and Searle 2002 |
| Denmark, Ribe, Nørre Farup | 55.3544 | 8.7328 | central Europe | GU563297 | DK02 |
| Denmark, Ribe, Nørre Farup | 55.3544 | 8.7328 | central Europe | GU563299 | DK09 |
| Denmark, Ribe, Mandø | 55.3072 | 8.6597 | central Europe | GU563298 | DK04 |
| England, Northumberland, Kielder | 55.2250 | -2.5750 | north Britain | AY167150 | Jaarola and Searle 2002 |
| England, Northumberland, Kielder | 55.2187 | -2.4448 | north Britain | GU563241 | K1 |
| England, Northumberland, Kielder | 55.2187 | -2.4448 | north Britain | GU563242 | K2 |
| England, Northumberland, Kielder | 55.2187 | -2.4448 | north Britain | GU563243 | K3 |
| England, Northumberland, Hexham | 54.9885 | -2.1109 | north Britain | GU563240 | NMS.Z.2009.101.490M |
| England, Cumbria, Brampton | 54.9687 | -2.6888 | north Britain | GU563244 | NMS.Z.2009.101.450M |
| England, Cumbria, Brampton | 54.9687 | -2.6888 | north Britain | GU563245 | NMS.Z.2009.101.451M |
| England, Northumberland, Newcastle | 54.9065 | -1.7442 | north Britain | FJ619759 | NMS.Z.2009.101.494M |
| England, Durham, Lanchester | 54.8176 | -1.7525 | north Britain | GU563247 | NMS.Z.2009.101.992M |
| England, Cumbria, Kendal | 54.3303 | -2.7396 | north Britain | GU563246 | NMS.Z.2009.101.690M |
| England, Cumbria, Kendal | 54.3303 | -2.7396 | north Britain | GU563248 | NMS.Z.2009.101.692M |
| England, Cumbria, Pennington | 54.1995 | -3.1496 | north Britain | GU563247 | NMS.Z.2009.101.657M |
| England, Walney, Biggar | 54.0773 | -3.2288 | north Britain | GU563248 | NMS.Z.2009.101.673 |
| England, Foulney | 54.0664 | -3.1536 | north Britain | GU563250 | NMS.Z.2009.101.643 |
| England, Walney, Wylock Marsh | 54.0657 | -3.2239 | north Britain | FJ619753 | NMS.Z.2009.101.675 |
| England, Sheep Island | 54.0641 | -3.2024 | north Britain | GU563248 | NMS.Z.2009.101.652 |
| England, Sheep Island | 54.0641 | -3.2024 | north Britain | GU563248 | NMS.Z.2009.101.658 |
| England, Sheep Island | 54.0641 | -3.2024 | north Britain | GU563248 | NMS.Z.2009.101.659 |
| England, Walney, South End | 54.0640 | -3.2116 | north Britain | GU563248 | NMS.Z.2009.101.667 |
| England, Piel Island | 54.0626 | -3.1733 | north Britain | GU563249 | NMS.Z.2009.101.691 |
| England, Walney | 54.0569 | -3.2083 | north Britain | GU563248 | NMS.Z.2009.101.646 |
| England, Yorkshire, Holgate | 53.9608 | -1.1053 | western | AY167170 | NMS.Z.2009.101.778M |
| England, Yorkshire, Holgate | 53.9608 | -1.1053 | western | AY167170 | NMS.Z.2009.101.779M |
| England, Yorkshire, York | 53.9468 | -1.0492 | north Britain | AY167192 | Jaarola and Searle 2002 |
| England, Yorkshire, Fulford | 53.9370 | -1.0556 | western | GU563253 | 238F2 |
| England, Yorkshire, Fulford | 53.9370 | -1.0556 | western | GU563253 | 278F1 |
| England, Yorkshire, Fulford | 53.9370 | -1.0556 | western | AY167170 | 421F6 |
| England, Yorkshire, York | 53.9354 | -1.0525 | western | AY167191 | Jaarola and Searle 2002 |
| England, Yorkshire, Bishopthorpe | 53.9193 | -1.0971 | western | GU563254 | Bi |
| England, Lancashire, Billington | 53.8123 | -2.4343 | north Britain | FJ619765 | NMS.Z.2009.101.1058M |
| England, Lancashire, Billington | 53.8123 | -2.4343 | north Britain | GU563252 | NMS.Z.2009.101.1059M |
| England, Lancashire, Billington | 53.8123 | -2.4343 | north Britain | FJ619765 | NMS.Z.2009.101.1067M |
| England, Lancashire, Billington | 53.8123 | -2.4343 | north Britain | FJ619765 | NMS.Z.2009.101.1068M |
| England, Lancashire, Billington | 53.8123 | -2.4343 | western | FJ619776 | NMS.Z.2009.101.1069M |
| England, Yorkshire, Wessenden | 53.5686 | -1.9260 | north Britain | GU563252 | NMS.Z.2009.101.1043M |
| England, Lancashire, Saddleworth | 53.5416 | -1.9577 | western | GU563251 | NMS.Z.2009.101.1044M |
| England, Derbyshire, Holme Moss | 53.5326 | -1.8808 | north Britain | FJ619764 | NMS.Z.2009.101.1045M |
| England, Lincolnshire, Epworth | 53.5269 | -0.8248 | western | AY167170 | NMS.Z.2009.101.658M |
| England, Lincolnshire, Epworth | 53.5269 | -0.8248 | western | AY167170 | NMS.Z.2009.101.659M |
| England, Lincolnshire, Epworth | 53.5269 | -0.8248 | western | FJ619771 | NMS.Z.2009.101.660M |
| England, Lancashire, Wigan | 53.5207 | -2.7104 | western | AY167170 | NMS.Z.2009.101.625M |
| England, Derbyshire, Crowden | 53.4877 | -1.8960 | north Britain | FJ619764 | NMS.Z.2009.101.1034M |
| England, Derbyshire, Crowden | 53.4877 | -1.8960 | western | FJ619775 | NMS.Z.2009.101.1037M |
| England, Cheshire, Wirrall | 53.2857 | -3.0064 | western | AY167170 | NMS.Z.2009.101.626M |
| England, Staffordshire, Longnor | 53.1874 | -1.8922 | western | GU563256 | NMS.Z.2009.101.896M |
| England, Derbyshire, Wensley | 53.1464 | -1.6082 | western | FJ619774 | NMS.Z.2009.101.968M |
| England, Staffordshire, Hardiwick | 53.0985 | -2.0926 | western | GU563255 | NMS.Z.2009.101.894M |
| England, Staffordshire, Hardiwick | 53.0985 | -2.0926 | western | FJ619773 | NMS.Z.2009.101.895M |
| England, Staffordshire, Weston Jones | 52.8112 | -2.3723 | western | AY167170 | NMS.Z.2009.101.902M |
| England, Norfolk, Grimston | 52.7645 | 0.5552 | western | FJ619768 | NMS.Z.2009.101.533M |
| England, Norfolk, Gayton | 52.7623 | 0.5699 | western | FJ619781 | NMS.Z.2009.101.534M |
| England, Shropshire, Morville | 52.5456 | -2.4748 | western | AY167170 | NMS.Z.2009.101.903M |
| England, Shropshire, Morville | 52.5456 | -2.4748 | western | AY167170 | NMS.Z.2009.101.904M |
| England, Shropshire, Colebatch | 52.4775 | -3.0056 | western | GU563257 | NMS.Z.2009.101.1910M |
| England, Warwickshire, Coventry | 52.3988 | -1.4576 | western | AY167170 | NMS.Z.2009.101.443M |
| England, Warwickshire, Coventry | 52.3988 | -1.4576 | western | GU563258 | NMS.Z.2009.101.447M |
| England, Warwickshire, Coventry | 52.3902 | -1.5606 | western | GU563259 | NMS.Z.2009.101.444M |
| England, East Sussex, Hailsham | 52.3748 | -1.9588 | western | GU563283 | NMS.Z.2009.101.543M |
| England, Worcestershire, Hopwood | 52.3748 | -1.9588 | western | AY167170 | NMS.Z.2009.101.594M |
| England, Worcestershire, Hopwood | 52.3748 | -1.9588 | western | FJ619770 | NMS.Z.2009.101.595M |
| England, Worcestershire, Hopwood | 52.3748 | -1.9588 | western | AY167170 | NMS.Z.2009.101.597M |
| England, Suffolk, Mildenhall | 52.3362 | 0.5246 | western | AY167170 | NMS.Z.2009.101.1079M |
| England, Suffolk, Desnage | 52.2765 | 0.5402 | western | AY167170 | NMS.Z.2009.101.537M |
| England, Suffolk, Gazely | 52.2473 | 0.5181 | western | AY167170 | NMS.Z.2009.101.535M |
| England, Gloucestershire, Little Witcombe | 51.8425 | -2.1248 | western | FJ619783 | NMS.Z.2009.101.781M |
| England, Gloucestershire, Epney | 51.7945 | -2.3291 | western | GU563255 | NMS.Z.2009.101.900M |
| England, Gloucestershire, Epney | 51.7945 | -2.3291 | western | GU563273 | NMS.Z.2009.101.901M |
| England, Gloucestershire, Woodmancote | 51.7887 | -2.0362 | western | GU563271 | NMS.Z.2009.101.898M |
| England, Gloucestershire, Woodmancote | 51.7887 | -2.0362 | western | GU563272 | NMS.Z.2009.101.899M |
| England, Oxfordshire, Shotover | 51.7515 | -1.1850 | western | AY167197 | Jaarola and Searle 2002 |
| England, Gloucestershire, Welsh Way | 51.7500 | -1.9290 | western | GU563269 | NMS.Z.2009.101.780M |
| England, Buckinghamshire, Great Missenden | 51.6978 | -0.6977 | western | FJ619772 | NMS.Z.2009.101.782M |
| England, Buckinghamshire, Hughenden | 51.6714 | -0.7505 | western | AY167170 | NMS.Z.2009.101.787M |
| England, Buckinghamshire, Ibstone | 51.6398 | -0.9176 | western | AY167170 | NMS.Z.2009.101.785M |
| England, Oxfordshire, Britwell Hill | 51.6181 | -1.0048 | western | AY167170 | NMS.Z.2009.101.786M |
| England, Wiltshire, Blundsden | 51.6060 | -1.8238 | western | AY167170 | NMS.Z.2009.101.784M |
| England, Oxfordshire, Russel's Water | 51.5926 | -0.9649 | western | GU563275 | NMS.Z.2009.101.783M |
| England, Gloucestershire, Horton | 51.5616 | -2.3317 | western | GU563270 | NMS.Z.2009.101.897M |
| England, Wiltshire, Grittenham | 51.5250 | -1.9250 | western | AY167193 | Jaarola and Searle 2002 |
| England, Gloucestershire, Bristol | 51.4945 | -2.6755 | western | GU563268 | NMS.Z.2009.101.449M |
| England, Kent, Dartford | 51.4687 | 0.2194 | western | AY167170 | MA1 |
| England, Kent, Dartford | 51.4687 | 0.2194 | western | AY167170 | MA3 |
| England, Kent, Dartford | 51.4687 | 0.2194 | western | AY167170 | MA5 |
| England, Wiltshire, Corsham | 51.4378 | -2.1884 | western | GU563274 | NMS.Z.2009.101.1111M |
| England, Surrey, Epsom | 51.3225 | -0.3152 | western | FJ619777 | NMS.Z.2009.101.1194M |
| England, Somerset, Chew Valley | 51.3159 | -2.6126 | western | FJ619766 | NMS.Z.2009.101.448M |
| England, Hampshire, Alice Holt | 51.1786 | -0.8522 | western | AY167170 | MA8 |
| England, Hampshire, Alice Holt | 51.1786 | -0.8522 | western | AY167170 | MA12 |
| England, Hampshire, Alice Holt | 51.1786 | -0.8522 | western | AY167170 | MA16 |
| England, Hampshire, Alton | 51.1547 | -0.9719 | western | GU563282 | NMS.Z.2009.101.437M |
| England, Hampshire, Avington | 51.0849 | -1.2447 | western | GU563281 | NMS.Z.2009.101.436M |
| England, East Sussex, Hailsham | 50.8442 | 0.3068 | western | FJ619769 | NMS.Z.2009.101.542M |
| England, Devon, Rewe | 50.7917 | -3.4750 | western | AY167170 | Jaarola and Searle 2002 |
| England, Isle of Wight, Havenstreet 2 | 50.7114 | -1.1997 | western | FJ619779 | NMS.Z.2009.101.1045 |
| England, Dorset, Dorchester | 50.7088 | -2.4404 | western | GU563278 | NMS.Z.2009.101.467M |
| England, Dorset, Dorchester | 50.7088 | -2.4404 | western | FJ619767 | NMS.Z.2009.101.468M |
| England, Isle of Wight, Thorley | 50.6896 | -1.4733 | western | GU563279 | NMS.Z.2009.101.1041 |
| England, Isle of Wight, Thorley | 50.6896 | -1.4733 | western | AY167170 | NMS.Z.2009.101.1042 |
| England, Isle of Wight, Thorley | 50.6896 | -1.4733 | western | GU563280 | NMS.Z.2009.101.1043 |
| England, Isle of Wight, Thorley | 50.6896 | -1.4733 | western | GU563279 | NMS.Z.2009.101.1044 |
| England, Dorset, Broadmayne | 50.6730 | -2.3976 | western | FJ619777 | NMS.Z.2009.101.466M |
| England, Devon (south) | 50.4276 | -3.6908 | western | GU563276 | 42SD |
| England, Devon (south) | 50.4276 | -3.6908 | western | AY167170 | 43SD |
| England, Devon (south) | 50.4276 | -3.6908 | western | GU563277 | 44SD |
| England, Cornwall, Newquay | 50.4131 | -5.0937 | western | FJ619780 | Co |
| Finland, Lapland, Tornio | 65.8500 | 24.1667 | eastern | AY167198 | Jaarola and Searle 2002 |
| Finland, Kainuu, Kuhmo | 64.1167 | 29.5167 | eastern | AY167205 | Jaarola and Searle 2002 |
| Finland, Southern Ostrobothnia, Lapua | 62.9833 | 22.9833 | eastern | AY167199 | Jaarola and Searle 2002 |
| Finland, South Karelia, Luumäki | 60.9167 | 27.6333 | eastern | AY167169 | Jaarola and Searle 2002 |
| Finland, Tavastia, Loppi | 60.7167 | 24.4333 | eastern | AY167195 | Jaarola and Searle 2002 |
| Finland, Tavastia, Loppi | 60.7167 | 24.4333 | eastern | AY167196 | Jaarola and Searle 2002 |
| Finland, Åland, Mellanön | 60.2500 | 19.5000 | eastern | AY167173 | Jaarola and Searle 2002 |
| Finland, Åland, Mellanön | 60.2500 | 19.5000 | eastern | AY167173 | Jaarola and Searle 2002 |
| France, Picardie, Abbeville | 50.1097 | 1.8278 | western | GU563287 | F182C7 |
| France, Picardie, Abbeville | 50.0833 | 1.5667 | western | GU563289 | F100C3 MA527 |
| France, Picardie, Abbeville | 50.0833 | 1.5667 | western | GU563289 | F131C3 MA543 |
| France, Île-de-France, Conches-sur-Gondoire | 48.8558 | 2.7159 | western | GU563284 | MNHN.2003.018 |
| France, Bretagne, Trebeurden | 48.7500 | -3.5667 | France | GU563290 | MA706 |
| France, Bretagne, St Malo | 48.6482 | -2.0261 | France | GU563288 | F264E1 MA462 |
| France, Bretagne, St Malo | 48.6482 | -2.0261 | France | GU563288 | F308E1 MA466 |
| France, Bretagne, St Malo | 48.6482 | -2.0261 | France | GU563288 | F309E1 MA467 |
| France, Bretagne, Gourin | 48.1391 | -3.6087 | France | GU563286 | FV1 |
| France, Franche-Comté, Etrabonne | 47.2335 | 5.7423 | western | GU563285 | MNHN.1999.582 |
| France, Doubs, Combe D'Orgeval | 46.9167 | 6.1833 | western | AY167188 | Jaarola and Searle 2002 |
| France, Doubs, Combe D'Orgeval | 46.9167 | 6.1833 | France | AY167189 | Jaarola and Searle 2002 |
| Germany, Schleswig-Holstein, Katinger Watt | 54.2831 | 8.8403 | central Europe | GU563294 | D29 |
| Germany, Schleswig-Holstein, Katinger Watt | 54.2831 | 8.8403 | central Europe | GU563295 | D30 |
| Germany, Schleswig-Holstein, Katinger Watt | 54.2831 | 8.8403 | central Europe | GU563293 | D31 |
| Germany, Schleswig-Holstein, Katinger Watt | 54.2831 | 8.8403 | central Europe | GU563296 | D32 |
| Germany, Schleswig-Holstein, Katinger Watt | 54.2831 | 8.8403 | central Europe | GU563296 | D33 |
| Germany, Schleswig-Holstein, Katinger Watt | 54.2831 | 8.8403 | central Europe | GU563296 | D34 |
| Germany, Niedersachsen, Emden | 53.3664 | 7.2133 | central Europe | GU563292 | D16 |
| Germany, North Rhine-Westphalia, Bonn | 50.7333 | 7.1000 | western | AY167210 | Jaarola and Searle 2002 |
| Lithuania, Vilnius | 54.6667 | 25.3167 | central Europe | AY167176 | Jaarola and Searle 2002 |
| Lithuania, Vilnius | 54.6667 | 25.3167 | eastern | AY167177 | Jaarola and Searle 2002 |
| Lithuania, Vilnius | 54.6667 | 25.3167 | eastern | AY167178 | Jaarola and Searle 2002 |
| Luxembourg, Luxembourg | 49.6100 | 6.1296 | western | GU563291 | LX13 |
| Netherlands, Gelderland, Heteren | 51.9667 | 5.7500 | western | AY167183 | Jaarola and Searle 2002 |
| Norway, Finnmark, Kvalsund, Sennalandet | 70.2800 | 24.0800 | eastern | KF218870 | Berg.005913 B.5486 * |
| Norway, Finnmark, Kvalsund, Sennalandet | 70.2800 | 24.0800 | eastern | KF218872 | Berg.005914 B.5487 * |
| Norway, Finnmark, Kvalsund, Sennalandet | 70.2800 | 24.0800 | eastern | KF218867 | Berg.005917 B.5491 * |
| Norway, Finnmark, Kvalsund, Sennalandet | 70.2800 | 24.0800 | eastern | KF218868 | Berg.005919 B.5494 * |
| Norway, Finnmark, Sør-Varanger, Korpfjellet | 69.5667 | 30.8500 | eastern | KF218871 | Berg.006727 B.7266 * |
| Norway, Finnmark, Sør-Varanger, Svanvik | 69.4565 | 30.0431 | eastern | KF218873 | Berg.006728 B.7268 * |
| Norway , Troms, Balsfjord, Malangen | 69.3522 | 18.8475 | eastern | KF218869 | Berg.002561 * |
| Norway, Møre og Romsdal, Aure, Tjeldbergodden | 63.4100 | 8.6800 | Scandinavia | KF218852 | Berg.006732 B.7273 * |
| Norway, Møre og Romsdal, Aure, Tjeldbergodden | 63.4100 | 8.6800 | Scandinavia | KF218859 | Berg.006733 B.7275 * |
| Norway, Møre og Romsdal, Aure, Tjeldbergodden | 63.4100 | 8.6800 | Scandinavia | KF218859 | Berg.006734 B.7276 * |
| Norway, Møre og Romsdal, Aure, Tjeldbergodden | 63.4100 | 8.6800 | Scandinavia | KF218859 | Berg.006735 B.7277 * |
| Norway, Sogn og Fjordane, Lærdal, Maristuen | 61.1000 | 8.0200 | Scandinavia | KF218855 | Berg.007242 * |
| Norway, Sogn og Fjordane, Lærdal, Maristuen | 61.1000 | 8.0200 | Scandinavia | KF218857 | Berg.007252 * |
| Norway, Sogn og Fjordane, Lærdal, Maristuen | 61.1000 | 8.0200 | Scandinavia | KF218857 | Berg.007253 * |
| Norway, Hordaland, Vaksdal, Ekse | 60.8276 | 6.2439 | Scandinavia | KF218866 | Berg.005309 B.3344 * |
| Norway, Hordaland, Vaksdal, Ekse | 60.8276 | 6.2439 | Scandinavia | KF218866 | Berg.005310 B.3309 * |
| Norway, Hordaland, Askøy, Herdla | 60.5674 | 4.9531 | Scandinavia | KF218862 | Berg.005541 * |
| Norway, Hordaland, Bergen, Åsane | 60.4826 | 5.3326 | Scandinavia | KF218865 | Berg.005225 * |
| Norway, Hordaland, Vaksdal, Lavik | 60.4372 | 5.1775 | Scandinavia | KF218851 | Berg.005430 B.3389 * |
| Norway, Hordaland, Vaksdal, Lavik | 60.4372 | 5.1775 | Scandinavia | KF218860 | Berg.005431 B.3390 * |
| Norway, Hordaland, Vaksdal, Lavik | 60.4372 | 5.1775 | Scandinavia | KF218851 | Berg.005432 B.3391 * |
| Norway, Hordaland, Bergen, Eidsvåg | 60.4364 | 5.3197 | Scandinavia | KF218864 | Berg.006729 B.7269 * |
| Norway, Hordaland, Eidfjord, Øvre Eidfjord | 60.4238 | 7.1289 | Scandinavia | KF218853 | Berg.006731 B.7271 * |
| Norway, Hordaland, Kvamsskogen, Kvam | 60.3846 | 6.1982 | Scandinavia | KF218861 | Berg.005224 * |
| Norway, Hordaland, Bergen, Fjøsanger | 60.3510 | 5.3167 | Scandinavia | KF218863 | Berg.005542 * |
| Norway, Hordaland, Bergen, Dortledhaugen | 60.3029 | 5.3072 | Scandinavia | KF218858 | Berg.006736 B.7278 * |
| Norway, Hordaland, Bergen, Kalandseidet | 60.2788 | 5.4218 | Scandinavia | KF218854 | Berg.006730 B.7269 * |
| Norway, Hedmark, Varaldskogen | 60.1667 | 12.4333 | Scandinavia | AY167202 | Jaarola and Searle 2002 |
| Norway, Akershus, Ås | 59.6669 | 10.8053 | Scandinavia | KF218856 | Berg.004366 * |
| Poland, Darżlubie | 54.7033 | 18.3231 | central Europe | KF218884 | MRI.PAS.10325 * |
| Poland, Darżlubie | 54.7033 | 18.3231 | central Europe | KF218884 | MRI.PAS.10319 * |
| Poland, Darżlubie | 54.7033 | 18.3231 | central Europe | KF218885 | MRI.PAS.10273 * |
| Poland, Stary Kraków | 54.4386 | 16.6161 | central Europe | KF218926 | MRI.PAS.13019 * |
| Poland, Żytkiejmy | 54.3478 | 22.6833 | central Europe | KF218949 | MRI.PAS.2551 * |
| Poland, Żytkiejmy | 54.3478 | 22.6833 | central Europe | KF218949 | MRI.PAS.2136 * |
| Poland, Żytkiejmy | 54.3478 | 22.6833 | central Europe | KF218948 | MRI.PAS.2207 * |
| Poland, Górowo Iławeckie | 54.2667 | 20.4833 | central Europe | KF218891 | MRI.PAS.6530 * |
| Poland, Górowo Iławeckie | 54.2667 | 20.4833 | central Europe | KF218892 | MRI.PAS.6101 * |
| Poland, Sierżno | 54.1210 | 17.4710 | central Europe | KF218923 | MRI.PAS.60190 * |
| Poland, Sierżno | 54.1210 | 17.4710 | central Europe | KF218923 | MRI.PAS.60191 * |
| Poland, Wigry | 54.0692 | 23.0938 | central Europe | KF218935 | MRI.PAS.18647 * |
| Poland, Pomorze | 54.0461 | 23.3626 | central Europe | KF218949 | MRI.PAS.27251 * |
| Poland, Pomorze | 54.0461 | 23.3626 | central Europe | KF218910 | MRI.PAS.27359 * |
| Poland, Pomorze | 54.0461 | 23.3626 | central Europe | KF218906 | MRI.PAS.27297 * |
| Poland, Bobolice | 53.9555 | 16.5866 | central Europe | KF218875 | MRI.PAS.14201 * |
| Poland, Bobolice | 53.9555 | 16.5866 | central Europe | KF218875 | MRI.PAS.14081 * |
| Poland, Płaska | 53.9045 | 23.2598 | central Europe | KF218914 | MRI.PAS.29489 * |
| Poland, Płaska | 53.9045 | 23.2598 | central Europe | KF218915 | MRI.PAS.29424 * |
| Poland, Płaska | 53.9045 | 23.2598 | central Europe | KF218916 | MRI.PAS.29319 * |
| Poland, Mikaszówka | 53.8906 | 23.3956 | central Europe | KF218949 | MRI.PAS.1223 * |
| Poland, Ryjewo | 53.8367 | 18.9586 | central Europe | KF218920 | MRI.PAS.43974 * |
| Poland, Ryjewo | 53.8367 | 18.9586 | central Europe | KF218920 | MRI.PAS.43934 * |
| Poland, Strzałowo | 53.7453 | 21.3650 | central Europe | KF218928 | MRI.PAS.39835 * |
| Poland, Strzałowo | 53.7453 | 21.3650 | central Europe | KF218928 | MRI.PAS.39836 * |
| Poland, Iława | 53.5964 | 19.5656 | central Europe | KF218894 | MRI.PAS.2851 * |
| Poland, Iława | 53.5964 | 19.5656 | central Europe | KF218895 | MRI.PAS.2342 * |
| Poland, Iława | 53.5964 | 19.5656 | central Europe | KF218896 | MRI.PAS.2681 * |
| Poland, Złocieniec | 53.5269 | 16.0122 | central Europe | KF218946 | MRI.PAS.68737 * |
| Poland, Złocieniec | 53.5269 | 16.0122 | central Europe | KF218947 | MRI.PAS.68691 * |
| Poland, Wierzchlas | 53.5180 | 18.1072 | central Europe | KF218933 | MRI.PAS.3191 * |
| Poland, Wierzchlas | 53.5180 | 18.1072 | central Europe | KF218934 | MRI.PAS.3074 * |
| Poland, Wierzchlas | 53.5180 | 18.1072 | central Europe | KF218934 | MRI.PAS.3112 * |
| Poland, Trzebieszki | 53.3619 | 16.6178 | central Europe | KF218931 | MRI.PAS.15082 * |
| Poland, Trzebieszki | 53.3619 | 16.6178 | central Europe | KF218932 | MRI.PAS.15381 * |
| Poland, Czarna Białostocka | 53.3029 | 23.2818 | central Europe | KF218881 | MRI.PAS.43825 * |
| Poland, Czarna Białostocka | 53.3029 | 23.2818 | central Europe | KF218882 | MRI.PAS.43732 * |
| Poland, Czarna Białostocka | 53.3029 | 23.2818 | central Europe | KF218883 | MRI.PAS.43881 * |
| Poland, Nowogród | 53.2264 | 21.8794 | central Europe | KF218912 | MRI.PAS.47283 * |
| Poland, Głusko | 53.0455 | 15.9442 | central Europe | KF218889 | MRI.PAS.70730 * |
| Poland, Głusko | 53.0455 | 15.9442 | central Europe | KF218890 | MRI.PAS.70770 * |
| Poland, Białowieża | 52.7000 | 23.8500 | central Europe | AY167185 | Jaarola and Searle 2002 |
| Poland, Bogdaniec | 52.6885 | 15.0679 | central Europe | KF218876 | MRI.PAS.71302 * |
| Poland, Bogdaniec | 52.6885 | 15.0679 | central Europe | KF218876 | MRI.PAS.71306 * |
| Poland, Bogdaniec | 52.6885 | 15.0679 | central Europe | KF218876 | MRI.PAS.71380 * |
| Poland, Starzyna | 52.5854 | 23.5311 | central Europe | KF218927 | MRI.PAS.36171 * |
| Poland, Starzyna | 52.5854 | 23.5311 | central Europe | KF218874 | MRI.PAS.36405 * |
| Poland, Łochów | 52.5317 | 21.7106 | central Europe | KF218911 | MRI.PAS.31818 * |
| Poland, Łochów | 52.5317 | 21.7106 | central Europe | KF218911 | MRI.PAS.31877 * |
| Poland, Nurzec | 52.4777 | 23.1734 | central Europe | KF218913 | MRI.PAS.31041 * |
| Poland, Bolewice | 52.3970 | 16.1187 | central Europe | KF218877 | MRI.PAS.77803 * |
| Poland, Rzepin | 52.3457 | 14.8326 | western | KF218921 | MRI.PAS.21229 * |
| Poland, Rzepin | 52.3457 | 14.8326 | central Europe | KF218922 | MRI.PAS.21407 * |
| Poland, Krosno Odrzańskie | 52.0573 | 15.0963 | central Europe | KF218903 | MRI.PAS.78963 * |
| Poland, Krosno Odrzańskie | 52.0573 | 15.0963 | central Europe | KF218904 | MRI.PAS.79195 * |
| Poland, Krosno Odrzańskie | 52.0573 | 15.0963 | central Europe | KF218903 | MRI.PAS.78933 * |
| Poland, Kryńszczak | 51.9906 | 22.3638 | central Europe | KF218905 | MRI.PAS.101163 * |
| Poland, Kryńszczak | 51.9906 | 22.3638 | central Europe | KF218906 | MRI.PAS.101202 * |
| Poland, Wschowa | 51.8056 | 16.3141 | central Europe | KF218938 | MRI.PAS.76937 * |
| Poland, Wschowa | 51.8056 | 16.3141 | central Europe | KF218938 | MRI.PAS.76977 * |
| Poland, Wschowa | 51.8056 | 16.3141 | central Europe | KF218938 | MRI.PAS.76957 * |
| Poland, Szprotawa | 51.5592 | 15.5362 | central Europe | KF218930 | MRI.PAS.80086 * |
| Poland, Szprotawa | 51.5592 | 15.5362 | central Europe | KF218888 | MRI.PAS.79952 * |
| Poland, Szprotawa | 51.5592 | 15.5362 | central Europe | KF218930 | MRI.PAS.80216 * |
| Poland, Wymiarki | 51.5089 | 15.0757 | central Europe | KF218939 | MRI.PAS.79721 * |
| Poland, Wymiarki | 51.5089 | 15.0757 | central Europe | KF218940 | MRI.PAS.79710 * |
| Poland, Wymiarki | 51.5089 | 15.0757 | central Europe | KF218941 | MRI.PAS.79671 * |
| Poland, Zagożdżon | 51.4817 | 21.4503 | central Europe | KF218942 | MRI.PAS.102006 * |
| Poland, Zagożdżon | 51.4817 | 21.4503 | central Europe | KF218942 | MRI.PAS.102005 * |
| Poland, Zagożdżon | 51.4817 | 21.4503 | central Europe | KF218943 | MRI.PAS.101810 * |
| Poland, Sobibór | 51.4750 | 23.6417 | central Europe | KF218924 | MRI.PAS.35997 * |
| Poland, Sobibór | 51.4750 | 23.6417 | central Europe | KF218925 | MRI.PAS.35909 * |
| Poland, Goszcz | 51.3959 | 17.4825 | central Europe | KF218893 | MRI.PAS.89218 * |
| Poland, Goszcz | 51.3959 | 17.4825 | central Europe | KF218888 | MRI.PAS.88997 * |
| Poland, Dębno | 51.3381 | 16.5234 | central Europe | KF218888 | MRI.PAS.89708 * |
| Poland, Dębno | 51.3381 | 16.5234 | central Europe | KF218886 | MRI.PAS.89627 * |
| Poland, Dębno | 51.3381 | 16.5234 | central Europe | KF218887 | MRI.PAS.89650 * |
| Poland, Rogalice | 50.9617 | 17.6088 | western | KF218917 | MRI.PAS.88705 * |
| Poland, Rogalice | 50.9617 | 17.6088 | central Europe | KF218918 | MRI.PAS.88708 * |
| Poland, Świętokrzyski PN | 50.9019 | 20.9625 | central Europe | KF218929 | MRI.PAS.37224 * |
| Poland, Świętokrzyski PN | 50.9019 | 20.9625 | central Europe | KF218929 | MRI.PAS.37280 * |
| Poland, Świętokrzyski PN | 50.9019 | 20.9625 | central Europe | KF218881 | MRI.PAS.37205 * |
| Poland, Lipa | 50.6956 | 22.0730 | central Europe | KF218908 | MRI.PAS.102392 * |
| Poland, Lipa | 50.6956 | 22.0730 | central Europe | KF218908 | MRI.PAS.102333 * |
| Poland, Lipa | 50.6956 | 22.0730 | central Europe | KF218909 | MRI.PAS.102240 * |
| Poland, Kosobudy | 50.6290 | 23.0745 | central Europe | KF218902 | MRI.PAS.102874 * |
| Poland, Kosobudy | 50.6290 | 23.0745 | central Europe | KF218900 | MRI.PAS.102644 * |
| Poland, Kosobudy | 50.6290 | 23.0745 | central Europe | KF218901 | MRI.PAS.102955 * |
| Poland, Zielona | 50.5474 | 18.9852 | central Europe | KF218944 | MRI.PAS.88117 * |
| Poland, Zielona | 50.5474 | 18.9852 | central Europe | KF218945 | MRI.PAS.88011 * |
| Poland, Zielona | 50.5474 | 18.9852 | central Europe | KF218945 | MRI.PAS.88099 * |
| Poland, Wojsław | 50.3833 | 21.5167 | central Europe | KF218936 | MRI.PAS.93573 * |
| Poland, Wojsław | 50.3833 | 21.5167 | central Europe | KF218937 | MRI.PAS.93566 * |
| Poland, Ruda Różaniecka | 50.3242 | 23.1892 | central Europe | KF218902 | MRI.PAS.93055 * |
| Poland, Ruda Różaniecka | 50.3242 | 23.1892 | central Europe | KF218919 | MRI.PAS.93130 * |
| Poland, Ruda Różaniecka | 50.3242 | 23.1892 | central Europe | KF218902 | MRI.PAS.93159 * |
| Poland, Kuźnia Raciborska | 50.2000 | 18.3114 | central Europe | KF218887 | MRI.PAS.88433 * |
| Poland, Kuźnia Raciborska | 50.2000 | 18.3114 | central Europe | KF218887 | MRI.PAS.88513 * |
| Poland, Kuźnia Raciborska | 50.2000 | 18.3114 | central Europe | KF218907 | MRI.PAS.88415 * |
| Poland, Bratkowice | 50.1143 | 21.8747 | central Europe | KF218878 | MRI.PAS.92149 * |
| Poland, Kobiór | 50.0603 | 18.9394 | central Europe | KF218897 | MRI.PAS.88349 * |
| Poland, Kobiór | 50.0603 | 18.9394 | central Europe | KF218898 | MRI.PAS.88351 * |
| Poland, Kobiór | 50.0603 | 18.9394 | central Europe | KF218899 | MRI.PAS.88338 * |
| Poland, Cisna | 49.2107 | 22.3293 | central Europe | KF218879 | MRI.PAS.40719 * |
| Poland, Cisna | 49.2107 | 22.3293 | central Europe | KF218880 | MRI.PAS.41149 * |
| Poland, Cisna | 49.2107 | 22.3293 | central Europe | KF218880 | MRI.PAS.41136 * |
| Romania, Rodrei Mts., Săpânta | 47.5000 | 24.5000 | central Europe | AY167172 | Jaarola and Searle 2002 |
| Romania, Rodrei Mts., Săpânta | 47.5000 | 24.5000 | central Europe | AY167172 | Jaarola and Searle 2002 |
| Romania, Valea Brazilor | 45.0333 | 24.6667 | central Europe | AY167172 | Jaarola and Searle 2002 |
| Russia, Yamal peninsula | 68.2500 | 70.0000 | eastern | AY167213 | Jaarola and Searle 2002 |
| Russia, Karelia, Pertozero | 62.0833 | 34.0000 | eastern | AY167153 | Jaarola and Searle 2002 |
| Russia, Karelia, Pertozero | 62.0833 | 34.0000 | eastern | AY167154 | Jaarola and Searle 2002 |
| Russia, Archangel region, Velsk | 61.0500 | 42.1000 | eastern | AY167166 | Jaarola and Searle 2002 |
| Russia, Sverdlovsk, Serov | 59.8333 | 60.3667 | eastern | AY167180 | Jaarola and Searle 2002 |
| Russia, Irkutsk, Bratsk | 56.2833 | 101.7000 | eastern | AY167171 | Jaarola and Searle 2002 |
| Russia, Moskva region, Chernogolovka | 56.0000 | 38.3667 | eastern | AY167165 | Jaarola and Searle 2002 |
| Russia, Novosibirsk | 55.0000 | 82.9167 | eastern | AY167149 | Jaarola and Searle 2002 |
| Russia, Novosibirsk | 55.0000 | 82.9167 | eastern | AY167156 | Jaarola and Searle 2002 |
| Russia, Novosibirsk | 55.0000 | 82.9167 | eastern | AY167157 | Jaarola and Searle 2002 |
| Russia, Novosibirsk | 55.0000 | 82.9167 | eastern | AY167175 | Jaarola and Searle 2002 |
| Russia, Penza region | 53.6667 | 42.2500 | eastern | AY167174 | Jaarola and Searle 2002 |
| Russia, Altai, Artybash | 51.7978 | 87.2262 | eastern | KF218950 | NMS.Z.1992.123.87 * |
| Russia, Altai, Artybash | 51.7978 | 87.2262 | eastern | KF218951 | NMS.Z.1992.123.88 * |
| Russia, Altai, Artybash | 51.7978 | 87.2262 | eastern | KF218952 | NMS.Z.1992.123.89 * |
| Scotland, Sutherland, Durness | 58.5845 | -4.7677 | north Britain | GU563195 | NMS.Z.2009.101.524M |
| Scotland, Sutherland, Durness | 58.5689 | -4.7407 | north Britain | FJ619760 | NMS.Z.2009.101.514M |
| Scotland, Sutherland, Loch Assynt | 58.1785 | -5.0406 | north Britain | GU563197 | NMS.Z.2009.101.87M |
| Scotland, Sutherland, Achmelvich | 58.1685 | -5.2983 | north Britain | GU563196 | NMS.Z.2009.101.86M |
| Scotland, Sutherland, Achmelvich | 58.1673 | -5.3067 | north Britain | GU563198 | NMS.Z.2009.101.92M |
| Scotland, Easter Ross, Balintore | 57.7529 | -3.9189 | north Britain | GU563206 | NMS.Z.2009.101.194M |
| Scotland, Wester Ross, Ardlair | 57.7330 | -5.5458 | north Britain | GU563204 | NMS.Z.2009.101.599M |
| Scotland, Wester Ross, Ardlair | 57.7330 | -5.5458 | north Britain | FJ619762 | NMS.Z.2009.101.600M |
| Scotland, Wester Ross, Ardlair | 57.7330 | -5.5458 | north Britain | FJ619762 | NMS.Z.2009.101.601M |
| Scotland, North Uist, Hougharry | 57.6086 | -7.5110 | north Britain | GU563200 | NMS.Z.2009.101.259 |
| Scotland, North Uist, Lochmaddy | 57.6055 | -7.1585 | north Britain | GU563199 | NMS.Z.2009.101.384 |
| Scotland, North Uist, Lochmaddy | 57.6055 | -7.1585 | north Britain | FJ619746 | NMS.Z.2009.101.385 |
| Scotland, Skye, Uig | 57.6027 | -6.3505 | north Britain | FJ619749 | NMS.Z.2009.101.350 |
| Scotland, Easter Ross, Strathpeffer | 57.5919 | -4.5362 | north Britain | GU563205 | NMS.Z.2009.101.200M |
| Scotland, North Uist, Loch an t-Seasgain | 57.5261 | -7.2510 | north Britain | FJ619746 | NMS.Z.2009.101.517 |
| Scotland, North Uist, Carinish | 57.5176 | -7.3233 | north Britain | FJ619746 | NMS.Z.2009.101.167 |
| Scotland, North Uist, Carinish | 57.5176 | -7.3233 | north Britain | FJ619746 | NMS.Z.2009.101.177 |
| Scotland, North Uist, Carinish | 57.5176 | -7.3233 | north Britain | FJ619746 | NMS.Z.2009.101.180 |
| Scotland, North Uist, Carinish | 57.5176 | -7.3233 | north Britain | FJ619746 | NMS.Z.2009.101.183 |
| Scotland, North Uist, Carinish | 57.5176 | -7.3233 | north Britain | FJ619746 | NMS.Z.2009.101.187 |
| Scotland, North Uist, Loch nan Garbh Chlachan | 57.5173 | -7.2447 | north Britain | FJ619746 | NMS.Z.2009.101.518 |
| Scotland, Inverness-shire, Morayhill | 57.5139 | -4.0850 | north Britain | GU563207 | NMS.Z.2009.101.150M |
| Scotland, Skye, Storr | 57.5113 | -6.1531 | north Britain | GU563203 | NMS.Z.2009.101.364 |
| Scotland, Easter Ross, North Kessock | 57.5041 | -4.2380 | north Britain | GU563205 | NMS.Z.2009.101.170M |
| Scotland, Skye, Loch Leathan | 57.4798 | -6.1822 | north Britain | FJ619746 | NMS.Z.2009.101.360 |
| Scotland, Inverness-shire, Kirkhill | 57.4541 | -4.4183 | north Britain | FJ619755 | NMS.Z.2009.101.253M |
| Scotland, Grampian, Binn of Fervie | 57.4250 | -2.0250 | north Britain | AY167181 | Jaarola and Searle 2002 |
| Scotland, Grampian, Leet Moss | 57.4083 | -2.3250 | north Britain | AY167182 | Jaarola and Searle 2002 |
| Scotland, Grampian, Leet Moss | 57.4083 | -2.3250 | north Britain | GU563209 | MJ2.63 |
| Scotland, Inverness-shire, Tomatin | 57.3357 | -3.9951 | north Britain | GU563208 | NMS.Z.2009.101.169M |
| Scotland, South Uist, Stilligarry | 57.3235 | -7.3708 | north Britain | FJ619746 | NMS.Z.2009.101.9 |
| Scotland, South Uist, Stilligarry | 57.3235 | -7.3708 | north Britain | GU563201 | NMS.Z.2009.101.10 |
| Scotland, South Uist, Stilligarry | 57.3235 | -7.3708 | north Britain | FJ619746 | NMS.Z.2009.101.14 |
| Scotland, South Uist, Stilligarry | 57.3235 | -7.3708 | north Britain | GU563201 | NMS.Z.2009.101.17 |
| Scotland, South Uist, Stilligarry | 57.3235 | -7.3708 | north Britain | GU563202 | NMS.Z.2009.101.80 |
| Scotland, Skye, Glen Varragill | 57.3154 | -6.2017 | north Britain | FJ619746 | NMS.Z.2009.101.356 |
| Scotland, Inverness-shire, Kyllachy | 57.3103 | -4.0087 | north Britain | GU563209 | NMS.Z.2009.101.202M |
| Scotland, South Uist, Loch nam Brae Ruaidh | 57.3000 | -7.3200 | north Britain | FJ619746 | NMS.Z.2009.101.383 |
| Scotland, South Uist, Askernish | 57.1876 | -7.4016 | north Britain | FJ619746 | NMS.Z.2009.101.250 |
| Scotland, Skye, Kinloch | 57.1747 | -5.7934 | north Britain | FJ619746 | NMS.Z.2009.101.288 |
| Scotland, Grampian, Tornaveen | 57.1433 | -2.6329 | north Britain | GU563210 | AB1 |
| Scotland, Grampian, Tornaveen | 57.1433 | -2.6329 | north Britain | GU563210 | AB2 |
| Scotland, Inverness-shire, Arisaig | 56.9343 | -5.8610 | north Britain | GU563213 | NMS.Z.2009.101.271M |
| Scotland, Inverness-shire, Arisaig | 56.9279 | -5.8669 | north Britain | FJ619756 | NMS.Z.2009.101.266M |
| Scotland, Inverness-shire, Arisaig | 56.9279 | -5.8669 | north Britain | GU563214 | NMS.Z.2009.101.276M |
| Scotland, Inverness-shire, Gairlochy | 56.9082 | -4.9792 | north Britain | GU563204 | NMS.Z.2009.101.242M |
| Scotland, Eigg, Blar Dubh | 56.9036 | -6.1455 | north Britain | GU563212 | NMS.Z.2009.101.460 |
| Scotland, Eigg, Allt Eas Chuthain | 56.8894 | -6.1422 | north Britain | FJ619750 | NMS.Z.2009.101.466 |
| Scotland, Muck, Port Mor | 56.8403 | -6.2385 | north Britain | GU563211 | NMS.Z.2009.101.554 |
| Scotland, Muck, Port Mor | 56.8377 | -6.2333 | north Britain | GU563211 | NMS.Z.2009.101.553 |
| Scotland, Muck, Port Mor | 56.8336 | -6.2214 | north Britain | GU563211 | NMS.Z.2009.101.551 |
| Scotland, Perthshire, Loch Crannach | 56.7885 | -3.5469 | north Britain | GU563222 | NMS.Z.2009.101.111M |
| Scotland, Inverness-shire, Laudale | 56.6759 | -5.6647 | north Britain | GU563204 | NMS.Z.2009.101.1165M |
| Scotland, Inverness-shire, Laudale | 56.6759 | -5.6647 | north Britain | GU563204 | NMS.Z.2009.101.1166M |
| Scotland, Lismore, Port Ramsay | 56.5527 | -5.4460 | north Britain | GU563216 | NMS.Z.2009.101.571 |
| Scotland, Lismore, Port Ramsay | 56.5527 | -5.4460 | north Britain | GU563217 | NMS.Z.2009.101.573 |
| Scotland, Lismore, Coeffin Road | 56.5329 | -5.4816 | north Britain | GU563217 | NMS.Z.2009.101.572 |
| Scotland, Mull, Fishnish | 56.5097 | -5.8388 | north Britain | GU563215 | NMS.Z.2009.101.849 |
| Scotland, Mull, Fishnish | 56.5097 | -5.8388 | north Britain | FJ619747 | NMS.Z.2009.101.850 |
| Scotland, Perthshire, Bankfoot | 56.5014 | -3.5140 | north Britain | AY167181 | NMS.Z.2004.212 |
| Scotland, Mull, Garmony | 56.4973 | -5.7758 | north Britain | FJ619747 | NMS.Z.2009.101.275 |
| Scotland, Mull, Craignure | 56.4691 | -5.6950 | north Britain | FJ619747 | NMS.Z.2009.101.276 |
| Scotland, Mull, Torosay | 56.4557 | -5.6937 | north Britain | FJ619747 | NMS.Z.2009.101.283 |
| Scotland, Argyll, Glen Lochy | 56.4095 | -4.8558 | north Britain | AY167181 | NMS.Z.2009.101.710M |
| Scotland, Argyll, Glen Lochy | 56.4095 | -4.8558 | north Britain | AY167181 | NMS.Z.2009.101.711M |
| Scotland, Argyll, Glen Lochy | 56.4095 | -4.8558 | north Britain | FJ619763 | NMS.Z.2009.101.712M |
| Scotland, Perthshire, Gask | 56.3583 | -3.6750 | north Britain | AY167190 | Jaarola and Searle 2002 |
| Scotland, Perthshire, Bridge of Earn | 56.3418 | -3.4090 | north Britain | AY167181 | NMS.Z.2009.101.101M |
| Scotland, Perthshire, Bridge of Earn | 56.3418 | -3.4090 | north Britain | AY167181 | NMS.Z.2009.101.1989M |
| Scotland, Perthshire, Strathallan | 56.3125 | -3.7377 | north Britain | GU563223 | NMS.Z.2009.101.1991M |
| Scotland, Seil | 56.2715 | -5.6275 | north Britain | GU563221 | NMS.Z.2009.101.834 |
| Scotland, Perthshire, Blackford | 56.2559 | -3.8013 | western | GU563224 | NMS.Z.2009.101.777M |
| Scotland, Luing | 56.2378 | -5.6436 | north Britain | GU563220 | NMS.Z.2009.101.832 |
| Scotland, Luing | 56.2378 | -5.6436 | north Britain | FJ619754 | NMS.Z.2009.101.833 |
| Scotland, Lunga | 56.2166 | -5.6917 | north Britain | GU563218 | NMS.Z.2009.101.829 |
| Scotland, Lunga | 56.2166 | -5.6917 | north Britain | GU563219 | NMS.Z.2009.101.830 |
| Scotland, Scarba | 56.1873 | -5.6792 | north Britain | GU563219 | NMS.Z.2009.101.831 |
| Scotland, Kinross, Cleish | 56.1570 | -3.4666 | north Britain | AY167181 | NMS.Z.2002.183.9 |
| Scotland, Kinross, Cleish | 56.1570 | -3.4666 | north Britain | AY167181 | NMS.Z.2002.183.10 |
| Scotland, Stirlingshire, Drymen | 56.0513 | -4.4582 | north Britain | FJ619757 | NMS.Z.2009.101.429M |
| Scotland, Midlothian, Edinburgh | 55.9418 | -3.2600 | north Britain | GU563239 | NMS.Z.2009.101.617M |
| Scotland, Bute (north) | 55.9203 | -5.1575 | north Britain | GU563237 | NMS.Z.2009.101.856 |
| Scotland, Midlothian, Currie | 55.8946 | -3.3096 | north Britain | GU563238 | NMS.Z.2009.101.422M |
| Scotland, Midlothian, Bonnyrigg | 55.8813 | -3.1077 | north Britain | FJ619758 | NMS.Z.2009.101.431M |
| Scotland, Jura, Craighouse | 55.8527 | -5.9432 | north Britain | GU563225 | NMS.Z.2009.101.828 |
| Scotland, Islay, Keills | 55.8440 | -6.1245 | north Britain | GU563227 | NMS.Z.2009.101.592 |
| Scotland, Bute (south) | 55.7694 | -5.0097 | north Britain | GU563237 | NMS.Z.2009.101.854 |
| Scotland, Bute (south) | 55.7694 | -5.0097 | north Britain | GU563236 | NMS.Z.2009.101.855 |
| Scotland, Islay, Laggan | 55.7431 | -6.2366 | north Britain | FJ619751 | NMS.Z.2009.101.492 |
| Scotland, Gigha, Tarbert | 55.7091 | -5.7328 | north Britain | FJ619752 | NMS.Z.2009.101.567 |
| Scotland, Arran, Cock of Arran | 55.7087 | -5.2407 | north Britain | GU563235 | NMS.Z.2009.101.1006 |
| Scotland, Gigha, Druimyeon More | 55.6928 | -5.7360 | north Britain | GU563229 | NMS.Z.2009.101.566 |
| Scotland, Kintyre, Ballure | 55.6904 | -5.6371 | north Britain | GU563232 | NMS.Z.2009.101.500M |
| Scotland, Gigha, PO Rd | 55.6791 | -5.7426 | north Britain | GU563228 | NMS.Z.2009.101.565 |
| Scotland, Islay, Kintra | 55.6587 | -6.2593 | north Britain | GU563226 | NMS.Z.2009.101.486 |
| Scotland, Islay, Kintra | 55.6587 | -6.2593 | north Britain | FJ619751 | NMS.Z.2009.101.487 |
| Scotland, Islay, Lagavulin | 55.6373 | -6.1202 | north Britain | GU563227 | NMS.Z.2009.101.595 |
| Scotland, Kintyre, Tayinloan | 55.6319 | -5.6682 | north Britain | GU563231 | NMS.Z.2009.101.499M |
| Scotland, Kintyre, Tayinloan | 55.6319 | -5.6682 | north Britain | GU563231 | NMS.Z.2009.101.504M |
| Scotland, Arran | 55.6259 | -5.1401 | north Britain | GU563234 | NMS.Z.2009.101.506 |
| Scotland, Arran, Pirates Cove | 55.6207 | -5.1333 | north Britain | GU563234 | NMS.Z.2009.101.507 |
| Scotland, Arran, Lamlash | 55.5260 | -5.1464 | north Britain | FJ619748 | NMS.Z.2009.101.345 |
| Scotland, Arran, Lamlash | 55.5260 | -5.1464 | north Britain | GU563233 | NMS.Z.2009.101.346 |
| Scotland, Kintyre, Southend | 55.3117 | -5.6417 | north Britain | FJ619761 | NMS.Z.2009.101.583M |
| Scotland, Kintyre, Southend | 55.3117 | -5.6417 | north Britain | GU563230 | NMS.Z.2009.101.586M |
| Sweden, Västerbottens Län, Umeå | 63.7667 | 20.2833 | eastern | AY167200 | Jaarola and Searle 2002 |
| Sweden, Västernorrlands Län, Gala | 63.3667 | 18.4500 | eastern | AY167201 | Jaarola and Searle 2002 |
| Sweden, Västernorrlands Län, Brunne | 62.6500 | 17.6333 | Scandinavia | AY167203 | Jaarola and Searle 2002 |
| Sweden, Gävleborgs Län, Lingbo | 61.0333 | 16.7000 | Scandinavia | AY167204 | Jaarola and Searle 2002 |
| Sweden, Stockholm | 59.4167 | 18.0833 | Scandinavia | AY167167 | Jaarola and Searle 2002 |
| Sweden, Stockholm | 59.4167 | 18.0833 | Scandinavia | AY167168 | Jaarola and Searle 2002 |
| Sweden, Västra Götaland Län, Hova | 58.8500 | 14.2167 | Scandinavia | AY167207 | Jaarola and Searle 2002 |
| Sweden, Östergötlands Län, Motala | 58.5333 | 15.0333 | Scandinavia | AY167206 | Jaarola and Searle 2002 |
| Sweden, Västra Götaland Län, Saltkällan | 58.4167 | 11.6833 | Scandinavia | AY167211 | Jaarola and Searle 2002 |
| Sweden, Blekinge Län, Nättraby | 56.1833 | 15.5167 | Scandinavia | AY167208 | Jaarola and Searle 2002 |
| Sweden, Blekinge Län, Nättraby | 56.1833 | 15.5167 | Scandinavia | AY167209 | Jaarola and Searle 2002 |
| Sweden, Skåne Län, Degeberga | 55.8167 | 14.0833 | Scandinavia | AY167203 | Jaarola and Searle 2002 |
| Switzerland, Vaud, Vallée de Joux | 46.5833 | 6.3500 | western | AY167158 | Jaarola and Searle 2002 |
| Switzerland, Vaud, Vallée de Joux | 46.5833 | 6.3500 | western | AY167159 | Jaarola and Searle 2002 |
| Wales, Anglesey, Llandona | 53.2879 | -4.1391 | western | GU563260 | NMS.Z.2009.101.1131M |
| Wales, Caernarvon, Bangor | 53.2183 | -4.1552 | western | FJ619784 | NMS.Z.2009.101.1130M |
| Wales, Anglesey, Capel Mawr | 53.2143 | -4.3661 | western | GU563262 | NMS.Z.2009.101.1129M |
| Wales, Anglesey, Capel Mawr | 53.2143 | -4.3661 | western | GU563261 | NMS.Z.2009.101.1298M |
| Wales, Anglesey, Capel Mawr | 53.2143 | -4.3661 | western | GU563263 | NMS.Z.2009.101.1299M |
| Wales, Anglesey, Malltraeth | 53.2019 | -4.3565 | western | FJ619786 | NMS.Z.2009.101.2001M |
| Wales, Anglesey, Malltraeth | 53.2019 | -4.3565 | western | GU563260 | NMS.Z.2009.101.2002M |
| Wales, Anglesey, Malltraeth | 53.2019 | -4.3565 | western | GU563260 | NMS.Z.2009.101.2003M |
| Wales, Caernarvon, Carmel | 53.0691 | -4.2507 | western | GU563264 | NMS.Z.2009.101.1135M |
| Wales, Caernarvon, Carmel | 53.0691 | -4.2507 | western | GU563264 | NMS.Z.2009.101.1241M |
| Wales, Dyfed, Aberystwyth | 52.3769 | -3.9964 | western | FJ619785 | NMS.Z.2009.101.1916M |
| Wales, Dyfed, Aberystwyth | 52.3769 | -3.9964 | western | FJ619778 | NMS.Z.2009.101.1917M |
| Wales, Dyfed, Lampeter | 52.0781 | -4.2122 | western | GU563265 | NMS.Z.2009.101.1918M |
| Wales, Carmarthen, Llanddowror | 51.7918 | -4.5824 | western | FJ619782 | NMS.Z.2009.101.557M |
| Wales, Pembroke, Tavernspite | 51.7721 | -4.6581 | western | FJ619782 | NMS.Z.2009.101.569M |
| Wales, Carmarthen, Pontyberem | 51.7599 | -4.1560 | western | GU563266 | NMS.Z.2009.101.546M |
| Wales, Carmarthen, Llannon | 51.7519 | -4.1484 | western | GU563267 | NMS.Z.2009.101.549M |
