# Supplementary material for: Land-Bridge Calibration of Molecular Clocks and the Post-Glacial Colonization of Scandinavia by the Eurasian Field Vole Microtus agrestis
Source: PLoS One. 2014 Aug 11;9(8):e103949. doi: 10.1371/journal.pone.0103949 (PMC4128820; doi:10.1371/journal.pone.0103949)
Supplement: Table S2 — Primers for cytochrome b amplification. Primers used for PCR amplification and sequencing of cytochrome b gene in Microtus agrestis (see Figure S1 for approximate positions of primers). (DOCX) [file pone.0103949.s003.docx]

**Table S2. Primers for cytochrome *b* amplification.**

Primers used for PCR amplification and sequencing of cytochrome *b* gene in *Microtus agrestis* (see Figure S1 for approximate positions of primers).

| Primer | Sequence (5’ to 3’) | Annealing Temp (°C) | Reference |
| --- | --- | --- | --- |
| L14727-SP | GACAGGAAAAATCATCGTTG | 52 | Jaarola & Searle (2002) |
| MicAg1R | TTCCTATGTTTCATGTTTCGATT | 52 | This study |
| MicAg2F | CCAACGGGGCCTCTATATTC | 57 | This study |
| MicAg2R | CATCTGAGTTTAAGCCAGTAGGG | 57 | This study |
| MicAg3Fn | AGTAGACAAGGCCACCCTCA | 52 | This study |
| MicAg3R | GTGCTCGTTGTTTTGAGGTG | 52 | This study |
| MicAgF4 | ACAAACTAGGCGGCGTCTTA | 54 | This study |
| MicAg4R | TGGTTTACAAGACCAGCGTAA | 54 | This study |
